# From closed to open: three dynamic states of membrane-bound cytochrome P450 3A4

**DOI:** 10.1007/s10822-025-00589-1

**Published:** 2025-03-17

**Authors:** Vera A. Spanke, Valentin J. Egger-Hoerschinger, Veronika Ruzsanyi, Klaus R. Liedl

**Affiliations:** 1 Department of Theoretical Chemistry, Universität Innsbruck, Innsbruck, Austria; 2 Department of Breath Research, Universität Innsbruck, Innsbruck, Austria

**Keywords:** Molecular dynamics simulations, Cytochrome P450 3A4, Tunnel, Membrane, Conformations

## Abstract

Cytochrome P450 3A4 (CYP3A4) is a membrane bound monooxygenase. It metabolizes the largest proportion of all orally ingested drugs. Ligands can enter and exit the enzyme through flexible tunnels, which co-determine CYP3A4’s ligand promiscuity. The flexibility can be represented by distinct conformational states of the enzyme. However, previous state definitions relied solely on crystal structures. We employed conventional molecular dynamics (cMD) simulations to sample the conformational space of CYP3A4. Five conformationally different crystal structures embedded in a membrane were simulated for 1 µs each. A Markov state model (MSM) coupled with spectral clustering (Robust Perron Cluster Analysis PCCA +) resulted in three distinct states: Two open conformations and an intermediate conformation. The tunnels inside CYP3A4 were calculated with CAVER3.0. Notably, we observed variations in bottleneck radii compared to those derived from crystallographic data. We want to point out the importance of simulations to characterize the dynamic behaviour. Moreover, we identified a mechanism, in which the membrane supports the opening of a tunnel. Therefore, CYP3A4 must be investigated in its membrane-bound state.

**Supplementary Information:**

The online version contains supplementary material available at 10.1007/s10822-025-00589-1.

## Introduction

Cytochrome P450 3A4 (CYP3A4) is a member of the cytochrome P450 superfamily and a monooxygenase with a heme in the active centre. It catalyses the oxidation of hydrophobic substances to more hydrophilic substances to facilitate their clearance. Thereby, it plays an important role in the metabolism of endogenous and exogenous substances [1]. CYP3A4 is involved in the detoxification of more than half of all orally ingested drugs [2]. However, it can cause drug-drug interactions [3] as well, and can turn harmless substances into toxic products by biotransformation [4]. Mammalian cytochrome P450 enzymes, including CYP3A4, are inserted partially into the membrane of the endoplasmic reticulum or mitochondria. A transmembrane helix is anchoring the enzyme inside the membrane and the globular part is partly immersed in the membrane [5]. CYP3A4 is mostly expressed in the liver and small intestine [6].

**Fig. 1:**
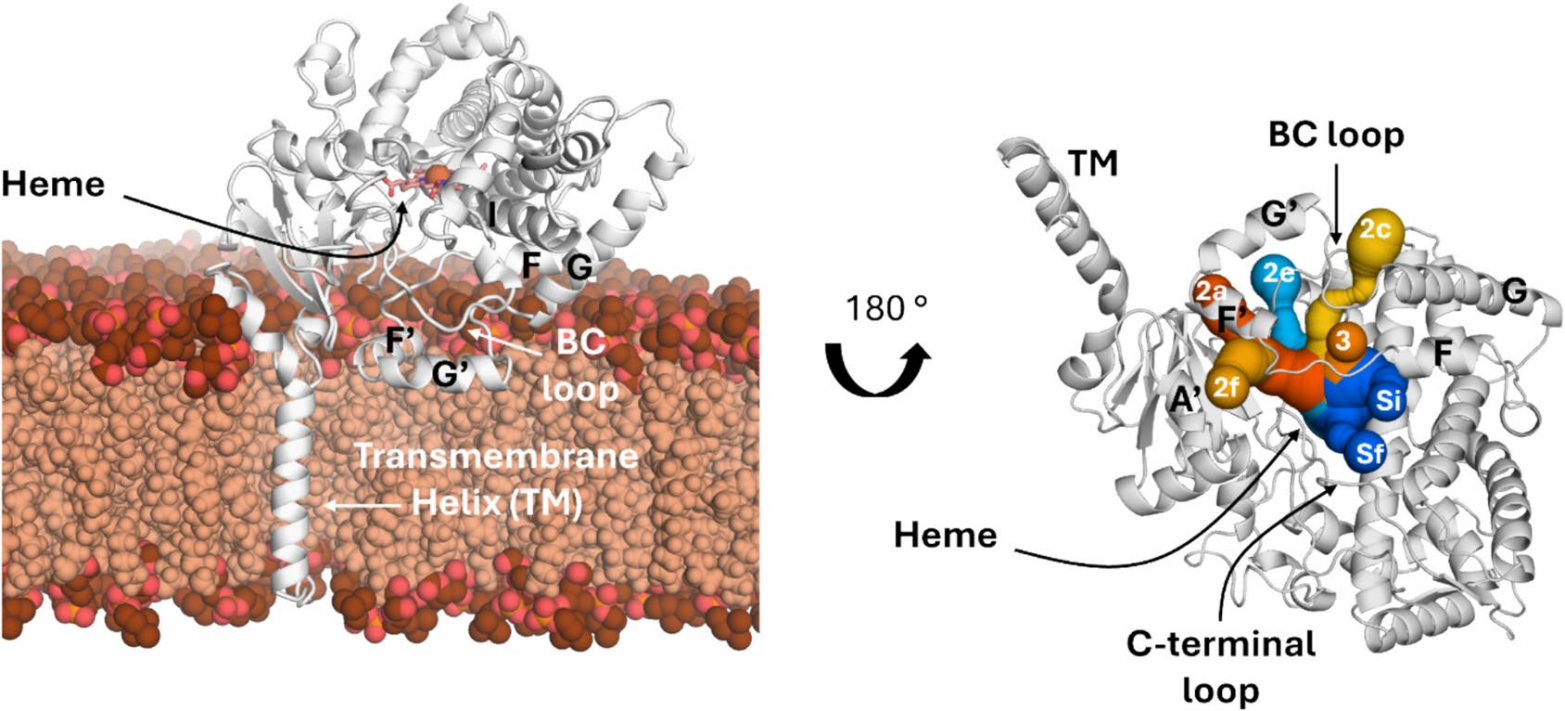
Structure of CYP3A4, its partial insertion into the membrane and the tunnels. The CYP3A4 enzyme is illustrated as a white cartoon representation, while the POPC membrane is shown as red and beige spheres. The transmembrane helix is completely inserted into the membrane and helices F′ and G′ are partly immersed in the membrane. The tunnels exit close to the F′, G′, G and F helix. The blue coloured tunnels (Si, Sf, 2e) are facing the solvent, while the orange and yellow tunnels (2f, 2c, 2a, 3) are exiting into the membrane. In our study, we divide tunnel S in tunnel Sf and Si, which is part of our results

CYP3A4 is known to be a promiscuous enzyme which can catalyse the biotransformation of various substrates. The catalytic centre of the enzyme, the heme, is in the centre of the globular protein. On top of the heme is the binding site, in which the substrates bind to be metabolized. Substrates and metabolites can only enter and exit the active centre via tunnels. The tunnels start close to the heme and pass by the following secondary structure elements: Helices G, F, G′, F′, I and A helices, as well as the FF′, GG′ and BC loop. Ludemann et al. described with the help of thermal motion pathway analysis three spatially and structurally distinct exit pathways in P450cam, called pathway 1,2 and 3 [7]. Following that, Cojocaru et al. calculated tunnels in crystal structures of the members of the P450 superfamily [8]. They clustered the tunnels and developed a nomenclature for the tunnels to compare them between members of the superfamily. In the original pathway 2 [7], they identified several distinct clusters of tunnels and named them according to the secondary structure elements, they pass (2e, 2f, 2a, 2c). Tunnel 3, one of the pathways identified by Ludemann, could not be observed in Cojocarus study. He also included the solvent tunnel (tunnel S) in his nomenclature, a proposed route for water to enter and exit the active site [9]. The subset of those tunnels, which will be further discussed in our study, is shown in Fig. 1.

A variety of experimental and computational studies has investigated the interplay of CYP flexibility, tunnel occurrence and openness, and ligand promiscuity. Ahalawat et al. observed in a multimicrosecond unbiased molecular dynamics (MD) simulation of P450cam, that Camphor prefers one single pathway [10]. However, different types of substrates can enter via separate tunnels, and educts and products do not have to pass through the same tunnel [11]. While caffeine in crystal structures is located outside of the surface close to the F′G′ helices and close to tunnels 2a and 2f [12], its metabolite 1,2,3-trimethyluric acid favours tunnel S to exit CYP3A4, as shown in a bias-exchange metadynamics study [13]. A hydrogen deuterium exchange mass spectrometry study described flexibility differences within CYP3A4 depending on distinct substrates or inhibitors entering and exiting the CYP, which indicates varying pathways for different molecules. The substrates and inhibitors had an increase in flexibility of the FF′ loop in common and showed flexibility differences in the BC region [14]. Therefore, it was claimed that the variety of tunnels codetermines the ligand promiscuity.

Moreover, many studies have evaluated the substrate promiscuity by analysing the structure and flexibility of the enzyme [15]. Skopalik et al. suggested after short MD simulations that the enzyme’s flexibility correlates with the ligand specificity. CYP3A4 showed higher flexibility and higher ligand promiscuity compared to CYP2A6 and CYP2C9, which exhibit both lower flexibility and ligand promiscuity [16]. In CYP enzymes different conformations, a result of high flexibility, are often summarized as open, intermediate and closed states. The conformational states are not only important for ligand promiscuity but also for the substrate metabolism process itself. Multiresolution molecular dynamics simulations in CYP 2B4 revealed that the open state supports ligand uptake and release while the closed state prolonged the residence time of the substrate inside the binding pocket [17]. Another multimicrosecond long adaptive sampled MD simulation examined the conformational changes in between closed, open and intermediate states in cytochrome P450cam. Movements of the B′ helix and FG loop determined the conformational states [18]. Another approach to demonstrate this flexibility, is to categorise the existing x-ray crystal structures into closed and open states, depending on the tunnel openness. Previous studies grouped them either depending on the presence of open tunnels [19] or in combination with the ligand sizes and their physicochemical properties, which dictate the volume of the binding site as well as the properties of the entrance tunnel [20]. Those studies were based on 9 and 24 RCSB Protein Data Bank (PDB) [21] structures, respectively. The clustering of existing pdb structures is a more generalized way to describe the conformational states, compared to a simulation based on a single CYP starting conformation. The pdb structures are missing the transmembrane helix, and in most of the structures are the FF′, HI and GH loops not resolved. A variety of distinct conformational states represents a high enzyme flexibility.

The membrane influences the flexibility of CYP3A4 as well. The helices F, F′, G′ and G, as well as their connecting loops FF′ and GG′ are highly flexible regions, which are in direct contact with the membrane (Fig. 1). Moreover, they are surrounding most of the tunnel entrances. Tunnel 2a, 2f, 3 and 4 are directly facing the membrane, as already shown before [22]. Hydrogen deuterium exchange mass spectrometry measurements showed that this region, helices F, F′, G′ and G and their connecting loops, is more rigid when inserted into a lipid nanodisc compared to an aqueous environment [23]. Another MD simulation study of CYP 2C9 inside a membrane supports this by observing the rigidification of the BC and FG region [24]. The membrane can also directly interact with the enzyme: a multimicrosecond long accelerated MD simulation described the unwinding of the G-helix as well as the disengagement of F241 from a hydrophobic interaction cluster [25]. Additionally, the membrane helps with ligand uptake. In the same accelerated MD simulation study, it was demonstrated that the hydrophobic testosterone accumulates inside the membrane and enters via a tunnel close to the F′–G′ helices facing the membrane [25]. Lastly, the orientation of the enzyme inside the membrane is determined by the conformational state, closed or open, of the enzyme [17].

To date, the closed and open states of CYP3A4, which depend on the openness of the tunnels, are not defined based on simulation data, but solely on crystal structures, without membrane effects considered. Moreover, the last definition based on 24 crystal structures is from year 2017. Furthermore, MD simulations conducted in membrane environments have been limited by their use of a single starting conformation, rather than capturing the full conformational ensemble. In this study, we evaluate the dynamic conformational ensemble and derive distinct states of CYP3A4 accounting to its natural membrane environment. Although our membrane model is a simple and less complex approximation of a natural membrane environment, we will explore the effect of the membrane on tunnel opening and closing.

In our study, we conducted a principal component analysis (PCA) on 39 PDB structures containing a resolved FF′ loop. From these structures, we selected five with unique conformations. These five conformations could be grouped into three different clusters. To capture the dynamics of CYP3A4, we employed conventional MD simulations of the selected five conformationally different crystal structures in an 1-Palmitoyl-2-oleoyl-sn-glycero-3-phosphocholin (POPC) membrane and simulated each complex for 1 µs. On the 5 µs of simulation data we applied Markov state modelling (MSM) and Robust Perron Cluster Analysis (PCCA +), which resulted in three distinct states. The tunnels and their bottleneck radii were calculated by CAVER3.0 [26]. It is a well-established tool to identify and characterize enzyme tunnels [27–31]. A bottleneck radius is the narrowest part of a tunnel and determines if a tunnel is open or closed. The states were analysed by their bottleneck radii distributions of the various tunnels, the interactions at the bottlenecks and the interactions with the membrane. Moreover, we analysed opening and closing correlations and tested the functionalities of the tunnel through ligand docking studies along the tunnels.

This paper defines three distinct states of a CYP3A4 enzyme in a membrane by employing conventional molecular dynamics simulations and points out the importance of taking the membrane interactions into account.

## Methods

To get an overview of the conformational landscape of the CYP 3A4 crystal structures in the RCSB Protein Data Bank (PDB) [21], we performed a principal component analysis (PCA) on 39 crystal structures with a resolved FG region (Online resource Fig. 11b, Table 5). We used the heavy atom cartesian coordinates of the aligned residues of the FG region (residues 202 to 258) and computed the PCA with the help of the pyEMMA tool (version 2.5.11) [32]. Following that, five CYP x-ray crystal structures (1TQN [33], 2V0M.A [34], 3NXU.A [35], 4NY4 [36], 6BD6 [37]) were taken without their ligands as starting structures for MD simulations from the RCSB Protein Data Bank. These structures were chosen as they span the currently known conformational space, depicted in our crystal structure PCA space. The missing loops (Online resource Fig. 10, Table 4) were modelled in MOE (Molecular Operating Environment, version 2018.08; Molecular Computing Group). AlphaFold2 (version 2.2.4) [38] was applied to the amino acid sequence ranging from residues 1 to 499, including mutation S18F, to model the transmembrane helix (residues 1 to 30). We checked for high predicted local distance difference test (pLDDT) values to trust in the modelled region. pLDDT values are an estimator for the modelling confidence in AlphaFold. They range from 0 to 100, with 100 indicating the highest confidence [39]. The last eight to eleven residues, varying in between the crystal structures, were not resolved in the crystal structures, therefore our structures end with the amino acid sequence DGT, which is resolved in 1TQN. The C-terminal end was capped with a N-methylamide and the N-terminal end with an acetylamide. The system with membrane consisted out of residues 1 to 501 while in the system without membrane the structure excluded the transmembrane helix and started at residue 25. To determine the flexibility within our modelled starting conformations, we aligned all structures to all heavy atoms in the modelled 1TQN structure, without membrane and solvent, and calculated the RMSD per residue with cpptraj (version 4.25.6), implemented in AmberTools21 [40]. All 10 structures (5 × with membrane, 5 × without membrane) were protonated to pH 7 in MOE using protonate3D [41].

To create the systems with membrane, all 5 starting structures were inserted into a POPC (1-Palmitoyl-2-oleoylphosphatidylcholine) membrane. We chose a simple, neutrally charged membrane system intentionally, to minimize the system complexity. The membrane was built with the Charmm-GUI bilayer builder (accessed December 2022) [42]. The orientation was adopted by orientations of proteins in membranes database (accessed December 2022) [43] (OPM). The initial length in X and Y direction was 155 Å and a TIP3P [44] water layer of 15 Å in Z direction was added on top and bottom. The 5 Charmm-GUI pdb format structures were converted into an AMBER-friendly pdb format that could be read with tleap by charmmlipid2amber.py, included in the AmberTools21 package [40].

Topologies for the 5 systems with and without membrane (10 in total) were prepared with the tleap tool in AmberTools21. Restrained electrostatic potential atomic partial charges (RESP-charges) as well as bond and angle parameter for the heme molecule, the coordinating cysteine and the iron, were extracted from the mcpb.py Amber tutorial[45]. MCPB.py is implemented in AmberTools and it generates Gaussian input files for geometry optimization (B3LYP/6-31G*), forcefield constant calculations (B3LYP/6-31G*), and Merz-Kollman RESP charge calculations, with a spin multiplicity of 6 and a sextet electronic structure for the metal site. The iron ion had a formal charge of 3 and the complex, consisting of the heme molecule, the iron and a deprotonated Cysteine, one of -2. The tutorial used gaussian09 (version Rev D.01) [46] to perform the calculations. Furthermore MCPB.py applies the Seminario method to generate forcefield parameters for the heme, deprotonated Cysteine and iron and it fits the RESP charges. Remaining protein parameters were derived from the AMBER 14SB forcefield [47] and the lipid17 [48] parameters were used for the membrane in systems with membrane. All systems without membrane were soaked in a cubic TIP3P [44] water box with minimum wall distance of 15 Å.

The systems without membrane were equilibrated using a multistep protocol [49]. The 5 systems with membrane were first minimized, then heated for 1 ps from 100 to 200 K with the Langevin thermostat under constant pressure with isotropic pressure scaling and a 10 Å cutoff for non-bonded interactions, minimized again, and heated further to 303.15 K under the same conditions as before in 10 ps. In CHARMM-GUI related studies are POPC membranes simulated at 303.15 K [50]. Subsequently the systems with membrane were equilibrated for 15.5 ns first with isotropic- then with anisotropic pressure scaling.

For each system (10 in total) we performed classical MD simulations for 1 µs in the NpT ensemble using AMBERs’ pmemd.cuda [51]. The systems without membrane were simulated with a Langevin thermostat [52] at 300 K, constant isotropic pressure at 1 bar [53], with SHAKE [54] on hydrogen bonds, to allow a timestep of 2 fs, and a 10 Å cutoff for non-bonded Lennard–Jones interactions. Long-ranged electrostatic interactions were treated with Particle-Mesh-Ewald, employing a uniform background charges to neutralize the system [55], while systems with membrane were simulated at 303.15 K (Langevin thermostat [52]), and anisotropic pressure scaling (Berendsen barostat [53]). In all simulations the backbone atoms of residues 25–499 were aligned to the first frame of the 1 µs cMD simulation starting from the PDB structure 1TQN without membrane.

To control the membrane integrity during the cMD simulations, we calculated the surface area per lipid for the lower and upper leaflet of the POPC bilayer in all five membrane simulations. To do this, we divided the box area in XY direction by the number of lipid molecules per leaflet. Moreover, we estimated the tilt angles in each membrane simulation, defined as the angle between the plane of the heme and the membrane normal. This parameter is critical for validating MD simulations against experimental measurements [6]. Using cpptraj, we calculated the membrane normal as the vector perpendicular to the least-squares best-fit plane through a selection of widely distributed phosphate atoms in the lower leaflet. Similarly, we determined the normal to the heme plane using the nitrogen atoms of the heme. The tilt angle was then calculated as 90° minus the absolute value of the angle between the two normal vectors (derived from their dot product).

The five individual simulations in each system were combined (2 × 5x 100,000 frames), and a stride of two was applied, resulting in two times 250,000 frames (50 frames/ns). Then, the obtained cMD trajectories for the systems with and without membrane were analysed separately with time-lagged independent component analysis [56] (tICA) of the backbone torsions of the bottleneck residues (Online resource Table 1), using the python library PyEMMA 2 [32] and employing a lag time of 70 ns for both systems. tICA serves as a dimensionality reduction method and is a technique to find the slowest-relaxing degrees of freedom. tICA shows high-autocorrelation linear combinations of the input degrees of freedom. The tICA space was then used for k-means clustering [57] to generate 500 microstates in both system, that build the basis for a Markov state model (MSM) [58]. The MSM was constructed employing a lag time of 40 ns for both systems, using PyEMMA 2. The lag times were chosen according to the implied timescale plot (Online resource Figs. 1 and 3), which shows an approximately constant behaviour of the estimated timescales at lag times over 40 ns for both systems. Following that, a PCCA + algorithm [59] was used to calculate macrostates. PCCA + is a spectral clustering method, which discretizes the sampled conformational space based on the eigenvectors of the transition matrix. The frames belonging to the same microstate were combined, resulting in a so-called state trajectory.

To calculate the tunnels, we saved every 50th frame of the three state trajectories (the state trajectories have in total 250 000 frames, 50 frames/ns) in a single pdb format, representing every nanosecond by a single MD simulation snapshot. The state trajectories are an individual combination of the simulations started from the five selected crystal structures, therefore the saved snapshots in pdb format are originating from those five individual cMD simulations. Eventually we saved 1001 (0.1% from 1TQN, 99.8% from 2V0M, 0.1% from 6BD6), 993 (0.1% from 1TQN, 99.9% from 3NXU) and 3008 (33.24% from 1TQN, 0.03% from 2V0M, 0.23% from 3NXU, 33.24% from 4NY4, 33.24% from 6BD6) structures in pdb format for the first, second and third state in systems with membrane, respectively; and in the systems without membrane 1001 (0.1% from 1TQN, 99.9% from 2V0M), 987 (0.2% from 1TQN, 99.8% from 3NXU) and 3015 (33.15% from 1TQN, 0.53% from 3NXU, 33.18% from 4NY4, 33.15% from 6BD6) structures in pdb format. We used CAVER 3.0 [26] to identify the tunnels in each pdb structure. The structure was represented by spheres of equal radii and a Voronoi diagram [60] was constructed. We selected the heme and the iron as the starting point for the tunnel calculations and excluded residue PHE304, ALA305, THR309 and CYS442 from the tunnel calculations. PHE304, ALA305 and THR309 protrude in the binding pocket directly on top of the heme and will adapt if a ligand enters the binding pocket. CYS 442 is coordinated with the heme iron from the opposite site of the binding pocket, but we neglected the tunnels approaching the heme from this site of the heme. We set the radius of the probe sphere to 0.9 Å, the default value [61], which limited the minimum radius within a tunnel to this radius. The radius of a shell sphere was defined by three times the largest radius in all calculated tunnels which regulates the bulk solvent vertices. The default value is set to two, but we selected a larger shell sphere radius to prevent the merging of two tunnels that are widely open near the surface, yet still separated from each other just below the surface. Here, we especially focused on the region in between the FF′ loop, A helices and the C-terminal loop. This region can open widely to a cleft, which we wanted to describe by a set of unmerged tunnels. All resulting tunnels, minimum cost pathways along the Voronoi graph, were clustered with an average linkage hierarchical clustering algorithm [62]. The benefit of Caver 3.0 is, that all calculated tunnels in every pdb structure are assigned to a cluster. Therefore, the tunnels in between the different pdb structures can be compared. We set the clustering threshold to 5 Å, the default value is 3.5 Å. Due to the bigger shell radius, we have obtained many tunnels, which differ only near the surface, but have a similar middle part. We chose the threshold to cluster tunnels, which have the same name according to Cojocaru’s nomenclature together, and to preserve the diverse tunnel set in the FF′ loop, A′ helix and C-terminal loop region [8]. Subsequently, we assigned to every cluster a tunnel type, guided by the nomenclature. The tunnels are solely clustered based on a pairwise distance matrix, considering the middle and end part of the tunnel path, as well as the clustering threshold.

Subsequently, the bottleneck of each tunnel was analysed. A bottleneck is the narrowest part of a tunnel. The bottleneck residues and the bottleneck radii were given by Caver 3.0. As soon as a tunnel was not calculated in a frame because it was closed, narrower than 0.9 Å, its bottleneck radius was set to 0 Å for the corresponding frame.

To further validate our systems, we investigated the insertion depth of tunnel openings (Online resource Fig. 14), defined by the bottleneck residues. Although bottleneck residues are not necessarily located at the surface of the CYP, it is a good measure to compare the insertion depth in varying tunnels and states. We calculated the distance in between the centre of mass of the bottleneck residues per tunnel and the plane spanned by the nitrogen atoms in the upper membrane leaflet. The apolar part of the membrane is described by the Cα atom of the oleic acid ester, in both the upper and lower leaflet.

After analysing the bottleneck radii residues given by Caver3.0, the interaction contacts in between selected bottleneck residues were calculated by getContacts [63], for systems with and without membrane. Each calculation was performed on each state trajectory, using every 50th frame for getContacts. The selected residues for the getContacts analysis are listed in online resource Table 2.

To analyse the effects of the membrane on tunnel opening and closing we calculated the native contacts, using the implementation in cpptraj [64] on each of the above-mentioned samples from the system with membrane. Residues 200 to 250, part of the F, F′, G′ and G helices, were selected and the contacts to all membrane molecules within 10 Å of the selected residues were determined. To compare the interactions in between the states, we calculated the relative membrane interaction time. This is the number of frames with a membrane interaction divided by the total number of frames in the given states *100%.

The flexibility of the enzyme within the state trajectories was calculated with the python package X-Entropy [65]. It calculates the entropy of the dihedral angle distribution within the state trajectory. We use dihedral entropy to measure the flexibility instead of root mean square deviations, because the dihedral entropy is alignment independent.

To assess the functionality of the identified tunnels, we used CaverDock (version 1.2) [66]. A tunnel is only functional, if a molecule can pass through the tunnel. Not only a narrow bottleneck is preventing the passage, but also a hydrophobic environment in case of a hydrophilic molecule or the other way round. CaverDock takes a tunnel and divides it into discs, creating a tunnel trajectory. Ligands are docked in the discs and with the help of the docking algorithm an energy profile along the tunnel trajectory is calculated. CaverDock employs the scoring function from AutoDock Vina to estimate the binding free energy [66, 67]. We applied CaverDock to 10 randomly selected, open tunnels for every tunnel type and state of the systems with membrane, resulting in 180 tunnels (tunnels 2e, 2a, Sf, Si, 3, 2f; three states; 10 randomly selected tunnels). Our ligands were caffeine, progesterone and water. Each of the three ligands was docked into the same set of tunnel conformations.

To evaluate correlated tunnel openings and closings we counted the number of frames in each state trajectory in which a pair of two different tunnel types opens together, closes together or frames in which only one of the two tunnel types is open, and the other one is closed. The counts were divided by the total number of frames in the corresponding state trajectory.

Graphics depicting the enzyme and membrane structure were generated with PyMOL(version 3.0.3) [68], while the other graphics were made with the python (version 3.7.13) package Matplotlib (version 3.5.3) [69]

## Results

We identified tunnel 2a, 2e, 2f, S, 2c and 3 in our simulations. The tunnels are named according to the nomenclature first postulated by Cojocaru et al. [8].Tunnel S is in our study separated into tunnel Si and tunnel Sf. The original tunnel S, described by Cojocaru et al., starts at the heme and exits the CYP close to the helices I and F and the FF′ loop. We found that the exit at the CYP surface can be bifurcated, caused by an interaction in between the I helix and the FF′ loop or F helix. In cases of a bifurcation at the exit, tunnel S is divided into two distinct tunnels, namely tunnel Si and tunnel Sf. The region including the exits of the solvent tunnels and tunnel 2f is prone to open widely. We tried to describe this region as detailed as possible by a set of tunnels; therefore, we chose a bigger shell radius to account for separations close to the surface. Tunnel Si exits closer to the I helix, compared to tunnel Sf, which leaves the enzyme closer to the F′ helix. Small changes close to the surface can already lead to a bifurcation to those two tunnels, describing this flexible region in more detail. Without the bifurcation, tunnel Si and Sf can share the same path through the enzyme. In the following sections, the opening and closing of those tunnels is analysed regarding the bottleneck radii, closing residues and membrane interactions. The analysis will focus on the membrane systems. Cojocaru et al. identified a tunnel 3 and a tunnel 4, tunnel 3 exiting in between the F and G helices and tunnel 4 exiting in the FG loop. In our work we were not able to assign the tunnels exiting in this region to either tunnel 3 or tunnel 4. As mentioned before, we increased the clustering threshold, which resulted in more dissimilar tunnels being merged into the same cluster. For every state, we identified only one cluster for tunnel 3 and 4, instead of the expected two clusters. Therefore, we combined both tunnels in this work, and tunnel 3 is a combination of tunnel 3 and 4.

## Validation of the (membrane) models

One benefit of working with CYP3A4 is, that there is a variety of x-ray structures in the PDB. Out of those 76 structures, we selected 39, which had a resolved FG region (Online resource Table 5). We focused on the FG region, because it is surrounding the tunnels exits and it is known to be flexible, meaning that most of the crystal structures have unresolved FF′ loops. The only loops, which are in less structures resolved are the GH and HI loops. However, those loops are not close to the tunnels and the active site on top of the heme (Online resource Fig. 10). Our selected x-ray crystal structures 1TQN, 2V0M, 3NXU, 4NY4 and 6BD6 are spread across the conformational space, spanned by PC1 and PC2 (Online resource 11b), based on the heavy atom cartesian coordinates in the FG region. The crystal structures can be divided into three groups: 6BD6 vs. 1TQN and 4NY4 vs. 3NXU and 2V0M. After modelling of the missing loops and transmembrane helix, we calculated the RMSD per residue in reference to the residues in 1TQN (Online resource Fig. 11a). It is not surprising that the FF′ loop shows the highest flexibility among the modelled structures following the modelled loops GH and HI. As already seen in the PCA (Online resource Fig. 11b), 3NXU and 2V0M are different from 1TQN, 4NY4 and 6BD6, especially in the FF′ loop. A major difference between those two groups is the orientation of F215. In 3NXU and 2V0M it is orientated towards the membrane, away from the active site. F213 is also differently orientated in between 1TQN, 4NY4, 6BD6 and 2V0M and 3NXU (Online resource Fig. 11c).

The transmembrane helix in every modelled membrane structure is predicted once with AlphaFold2. The pLDDT values for the helix range from 82.58 to 94.67. Only the N-terminal end, which is already outside of the lower leaflet has lower pLDDT values (Online resource 10b). The tilt angles are higher at the start of the simulation but relax to lower values during the simulations, ranging from 56.8° in 2V0M to 61.4° in 6BD6 (Online resource Fig. 12). Moreover, the surface area per lipid is constant, with an average area per lipid of 68.8 Å^2^ in the lower leaflet and 71.3 Å^2^ in the upper leaflet over all five membrane simulations (Online resource Fig. 13). In the upper leaflet is more area occupied by the protein, resulting in greater area per lipid values.

## Definition of states

**Fig. 2:**
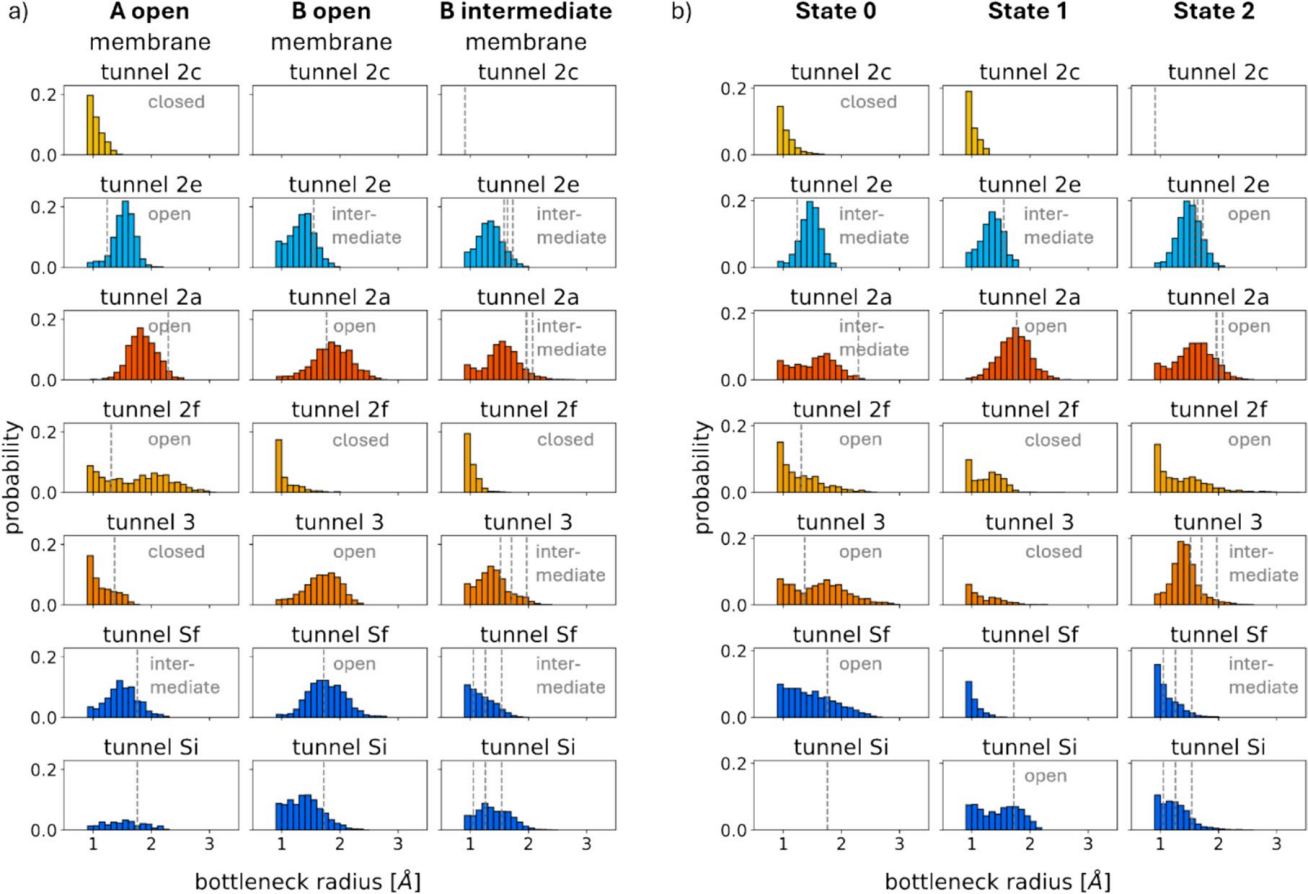
The distribution of bottleneck radii for each tunnel type across different states is shown for systems with a membrane (**a**) and systems without a membrane (**b**). Dashed lines indicate the bottleneck radii observed in the crystal structures used as starting conformations for the simulations. We labelled each tunnel with the assigned level of tunnel openness

In the systems with membrane the Robust Perron Cluster Analysis (PCCA +) of in total 5 µs MD simulation data resulted in three distinct states of CYP3A4. The third state has the highest population (Online resource Table 3). The states differ in how often the tunnels open (counts of each tunnel, in which the tunnel is open wider than 0.9 Å divided by the total number of structures extracted for each state), how wide they open (bottleneck radius in an open, wider than 0.9 Å, tunnel in a structure extracted from the state trajectory), and which tunnel is the widest and most often opened tunnel.. The distributions of the bottleneck radii are illustrated in Fig. 2. Tunnels 2e and 2a show minimal variation between states. Also, the solvent tunnels, consisting out of tunnel Si and Sf, exhibit a similar opening probability in between the states, if tunnel Si and Sf are treated equally and if only the wider opened tunnel represents the solvent tunnel. Whereas tunnels 2f and 3 exhibit significant differences. In the following we will call a tunnel “closed” in a certain state, if this tunnel does not open wider than 1.7 Å, the van-der-Waals radius of water [70]. Tunnel 2f is open in the first state and closed in the other states. It can merge with the solvent tunnels in the first state and create an even larger tunnel than described in Fig. 2. In contrast to this, tunnel 3 is closed in the first state but open in the other states. Based on these differences, the three states can be grouped into two categories: Group A (first state) and Group B (second and third state). The tunnels in the last state open less frequently and widely compared to the second state. Consequently, we will call the first state A_open, the second state B_open, and the last state B_intermediate. Tunnel 2c will not be further discussed due to its infrequent opening and small bottleneck radii.

The conformation of the 2V0M crystal structure is part of state A_open, the conformations of the other crystal structures fall within state B_intermediate (Online resource Fig. 1b). All simulations, except the simulation started from 4NY4, transition in between the states (Online resource Fig. 1d). The majority of state B_open originates from the 3NXU simulation.

The bottleneck radii calculated for the crystal structures deviate from those observed during the simulations. A striking example is tunnel 3 in state B_open. The original crystal structure 3NXU features a closed tunnel 3, while it opens during the simulation. In contrast, tunnel 2a is more widely open in the crystal structures (2V0M, 1TQN, 4NY4, 6BD6) compared to the simulations in the A_open and B_intermediate states. Tunnel 2e consistently maintains bottleneck radii below 2 Å and does not open as widely as the other tunnels.

Time-lagged independent component analysis (tICA) identifies collective degrees of freedom by their autocorrelation, correlating to the slowest protein movements. In Fig. 1b) given in our online resource, we show the two slowest tICs as a description of the sampled conformational space. Comparing the coverage of the combined conformational space of the simulations with membrane to the simulations without membrane reveals that they share similar minima (Online resource Fig. 2). An independent Markov state model (MSM) for the simulations without membrane identified three PCCA + states as well, similar to the simulations with membrane. The simulation of 2V0M defines the first state, the simulation started from 3NXU corresponds to the second state and the conformations of the other crystal structures fall within the last state. Thus, the states in the systems without a membrane closely resemble those in the membrane systems, although the simulations without a membrane transition between states more frequently.

In the systems without membrane, the most pronounced differences between the states are within tunnel 2f and 3, as in the simulations with membrane. However, these two tunnels no longer exhibit opposite opening behaviours. Tunnel 2f differs less extreme between the states and opens less frequently as wide as in simulations with membrane. Without membrane, there is no state in which all tunnels within the state are more closed than in the other states, like B intermediate in the systems with membrane. A potential intermediate state in the systems without membrane is state 1, but tunnel 2a and tunnel Si open wider than in state 0 and state 2 respectively and can be labelled as open. Therefore, a categorization into A_open, B_open and B_intermediate would not hold true anymore. The states in systems without membrane are not categorized into more open or closed states and are called states 0, 1 and 2. State 0 and 2 could be described as more open states. Simulations started from 4NY4 or 6BD6 led to either state B_intermediate in systems with membrane or to state 2 in systems without membrane. Surprisingly, state B_intermediate is a more closed state, while state 2 is a more open state. Therefore, the states in between the systems with and without membrane share similar backbone conformations, as they show similar minima in the tICA space. But despite that, the opening patterns of the tunnels change with and without membrane. Hence, the tunnels are heavily influenced by small sidechain shifts.

## Interaction patterns

**Fig. 3:**
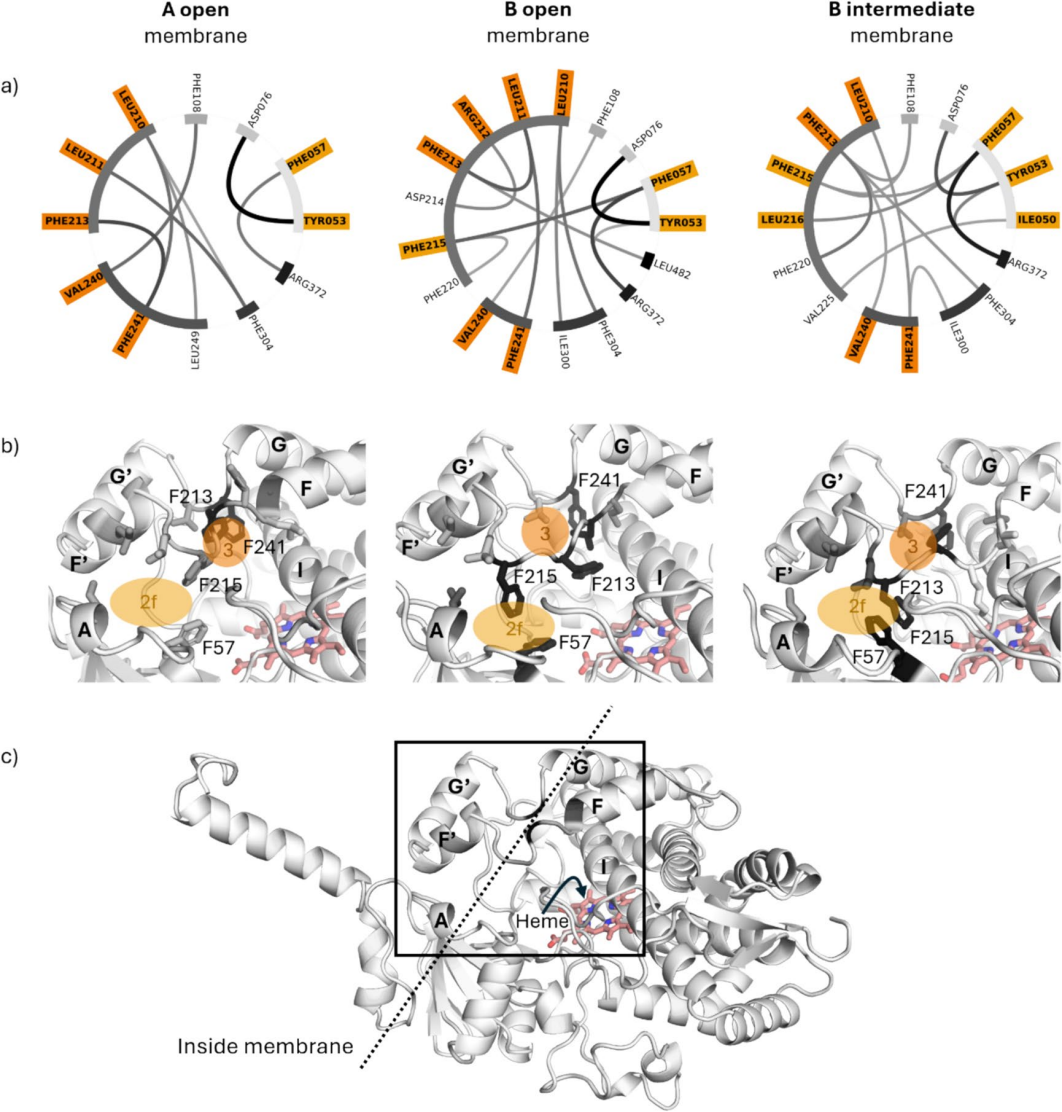
Interaction patterns of the bottleneck residues in tunnel 2f and 3 in state A_open, B_open and B_intermediate. **a** Each line represents an interaction, the thicker and darker the line, the more frequent this interaction is in the state trajectory. The disconnected grey border indicates which residues belong to the same secondary element of CYP3A4. The residues marked in colour are bottleneck residues of either tunnel 2f, in light orange, or tunnel 3, in darker orange. **b** Representative state structures. The darker the colour of the residues depicted in stick representation, the more they are interacting in the state trajectory. The orange circles describe the tunnel exits: light orange for tunnel 2f and dark orange for tunnel 3. **c** The cartoon representation of the CYP3A4, the membrane is not shown but indicated by the dashed line. The black rectangle marks the area, which is represented in more detail in (b)

To describe the differences between the states, we begin by analysing the varying interaction patterns. The most significant differences between group A and B are observed in the opposite opening behaviours of tunnels 2f and 3. Those tunnels vary not only in between the states within one system but also in between the systems with and without membrane. We calculated the interactions and the interaction frequencies for their bottleneck residues, as presented in Fig. 3. A bottleneck residue is defined as a residue which is lining the narrowest part of a tunnel, making it particularly susceptible to closing the tunnel. An interaction, which is closing the tunnel, is present in the closed state of the tunnel but not in the open.

In the case of tunnel 2f, it is closed by PHE57 and PHE215 or PHE57 and LEU216 as they start interacting in the states of B, in which tunnel 2f is closed. Other interactions that are unique to the closed states are located further from the tunnel and primarily serve to stabilize the closed state. Figure 3 displays the representative structures of the three states. The binding site, in which the substrates bind to be metabolized, is located directly above the heme, next to the I helix. In state A_open, where tunnel 2f is open, PHE215 and LEU216 are oriented outward, away from the binding site, and are too distant to interact with potential partners.

For tunnel 3, the main agonist for closing the tunnel is PHE213. In state A_open, where tunnel 3 is predominantly closed, PHE213 interacts with PHE241 to close the tunnel. In contrast, tunnel 3 is most open in state B_open, where PHE213 is positioned far from the tunnel. In state B_intermediate is PHE213 interacting with multiple other residues and the tunnel is only transiently blocked. The arrangement of these interactions is illustrated in Fig. 3.

## Membrane interactions

**Fig. 4:**
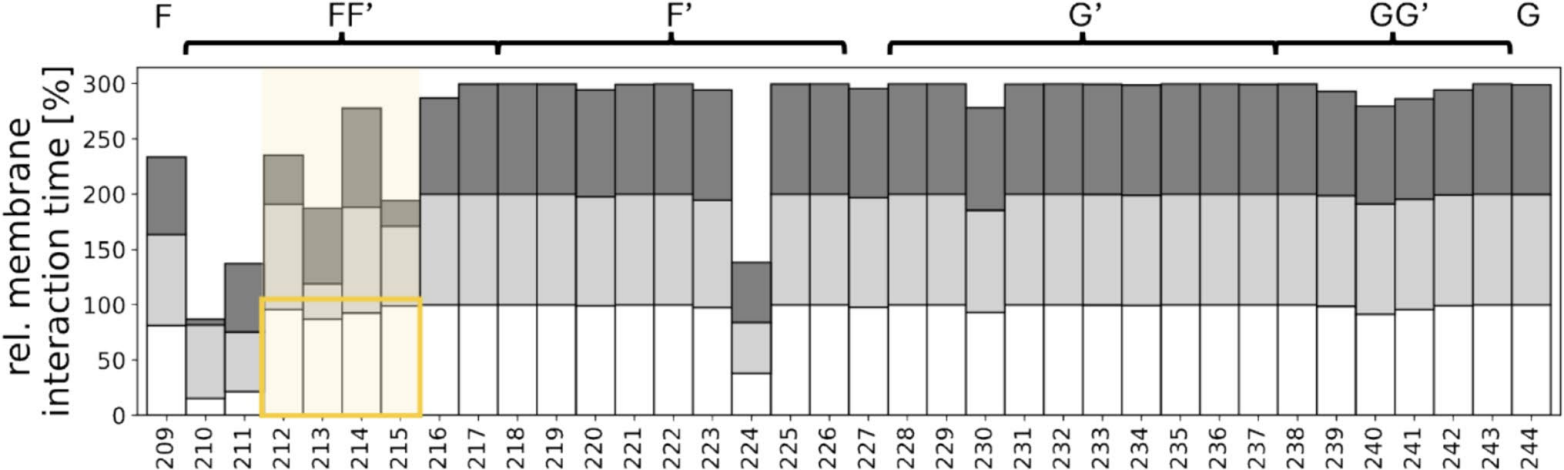
This figure displays the relative membrane interaction time for each residue of the FG region, with percentages stacked to show the interaction across all states. A value of 300% indicates that the corresponding residue interacts with the membrane in every frame and state. Residues are color-coded by state: white for A_open, light grey for B_open, and dark grey for B_intermediate. The orange area highlights residues in the FF′ loop. The secondary structure elements are plotted on top

CYP3A4 is partly immersed in the membrane and anchored by a transmembrane helix. The FF′ loop, the F′ helix, the G′ helix and the GG′ loop primarily interact with the membrane (Figs. 1 and 4). Most of the tunnel exits are located within this region. Consequently, the behaviour of all tunnels can be influenced by the membrane. As mentioned before, tunnel 2f and 3 are affected the most by the membrane: Their opening patterns differentiate the states of group A and B. The most notable differences in membrane interactions between states are observed in the FF′ loop (orange in Fig. 4). The FF′ residues (R212, F213, D214, F215, L216, D217, P218) are interacting nearly the entire simulation time of state A_open with the membrane. Tunnel 2f is open in state A_open and can be closed by interactions of residues in the A helices (F57) with residues in the FF′ loop (F215, L216), which are in contact with the membrane as well. Visual inspection of state A_open revealed that membrane lipids start to go inside tunnel 2f. In the states of group B the FF′ loop is not as strongly supported by the membrane as in state A_open.

Moreover, the membrane interactions can be used to indirectly measure how densely the binding site, above the heme, is packed. Residues, which are not interacting with the membrane tend to be oriented inward, pointing towards the binding site. State B_intermediate is characterized by smaller bottleneck radii and all residues of the FF′ loop in state B_intermediate are interacting less often with the membrane than in the other states. Especially ARG212, PHE215 and LEU216 appear to be buried inside the binding site.

We also analysed the effects of the membrane on the flexibility of CYP3A4 (Online resource Fig. 6). To do this, we calculated the dihedral entropy, which is alignment independent and represents local flexibility. Especially the FF′ loop becomes more rigid upon insertion into the membrane.

## Hydrophobic or hydrophilic tunnels

Tunnel 3, 2f and 2a are in every state closer to the membrane compared to the tunnels Sf, Si and 2e (Online Resource Fig. 14). Although tunnel 2a, like tunnels 2f and 3, is embedded in the membrane, it is lined with more charged residues. In contrast, tunnels 2f and 3 are clearly hydrophobic, while the solvent tunnels Sf and Si are the most polar tunnels, which are facing the solvent. Tunnel 2e is also exiting towards the solvent, but shows a similar physicochemical profile to tunnel 2a, a membrane tunnel (see Table 1).

**Table 1:** The bottleneck residues for each tunnel are categorized according to their physical characteristics

Tunnel	Hydrophobic	Neutral/polar	Charged
2a	P107, F108, F215, F220,	T224	D076, R106, E374
2e	P107, F108, I120, A121	N104, S119	R105, R106
2f	I50, F57, F215, L216, L221	Y53	
Sf	L211, L482,	Q484	K173, R212, E308
Si	L210, L211	T207, Y307	K208, R212, E308
3	L210, L211, F213, V240, F241		R212

## Ligand pathways and tunnel functionality

**Fig. 5:**
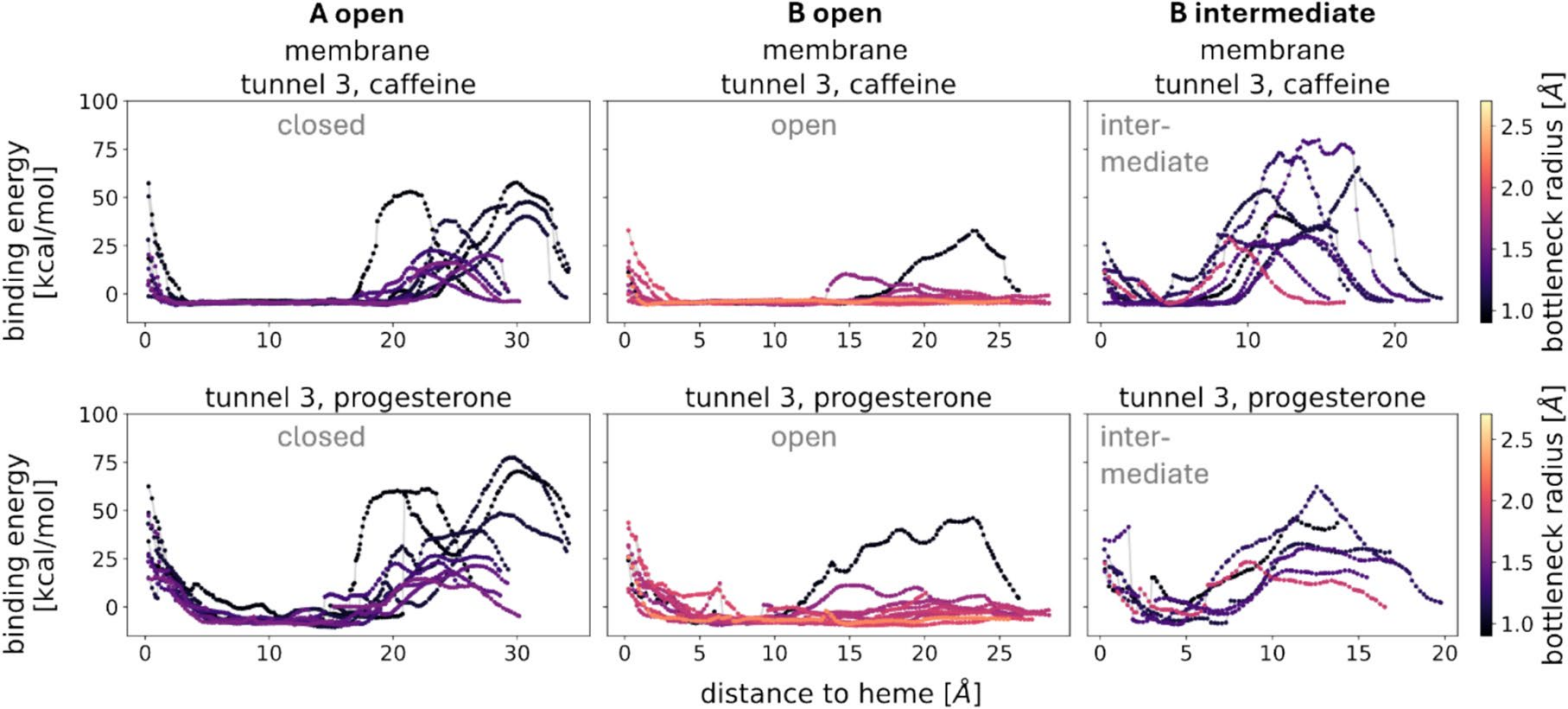
Energy profiles of caffein (top row) and progesterone (bottom row) within tunnel 3 in state A_open, B_open and B_intermediate. For each state, both ligands were docked into the same tunnels. Dark purple lines represent energy profiles for tunnels with small bottleneck radii, while light orange lines belong to tunnels with large bottleneck radii. The closed, open and intermediate labels are based on the bottleneck radii distributions in Fig. 2

To evaluate the functionality of open tunnels, we performed docking of three different ligands along randomly selected open conformations of each tunnel in every state of the systems with a membrane. The results are shown in Fig. 5 and in online resource Fig. 7. On one hand a high energy barrier prevents a ligand from passing through a specific tunnel and on the other hand low estimated binding free energies, below 0 kcal/mol, indicate, that the ligand can exit via the tunnel. In Fig. 5 tunnel 3 in state B_open exhibits for both ligands along the entire tunnel pathway estimated binding free energies below 0 kcal/mol, and both ligands can pass tunnel 3 easily in state B_open.

The first ligand tested was water, which encountered low energy barriers across all tunnels. The second ligand was caffeine, a planar hydrophobic molecule with polar groups. Caffeine is smaller than the third ligand, progesterone. In general, the tunnels exhibit lower energy barriers for caffeine compared to progesterone and both ligands pass more easily through tunnels with wider bottleneck radii. In tunnel 3 state B_intermediate and in tunnel Si in states A_open and B_open are conformations, that are sufficiently large opened (> 1.7 Å) but the docking result shows high energy barriers. Exemplary in Fig. 5 we show the docking results of both ligands in tunnel 3, in which the energy profile of progesterone demonstrates in general higher energy barriers compared to caffeine, although they are docked in the same tunnel conformations of tunnel 3. As mentioned before, in state B_intermediate is the energy barrier of the lightest coloured tunnel higher, than in tunnel conformations with comparable bottleneck radii in the other two states, A open and B open.

## Correlated opening and closing

**Fig. 6:**
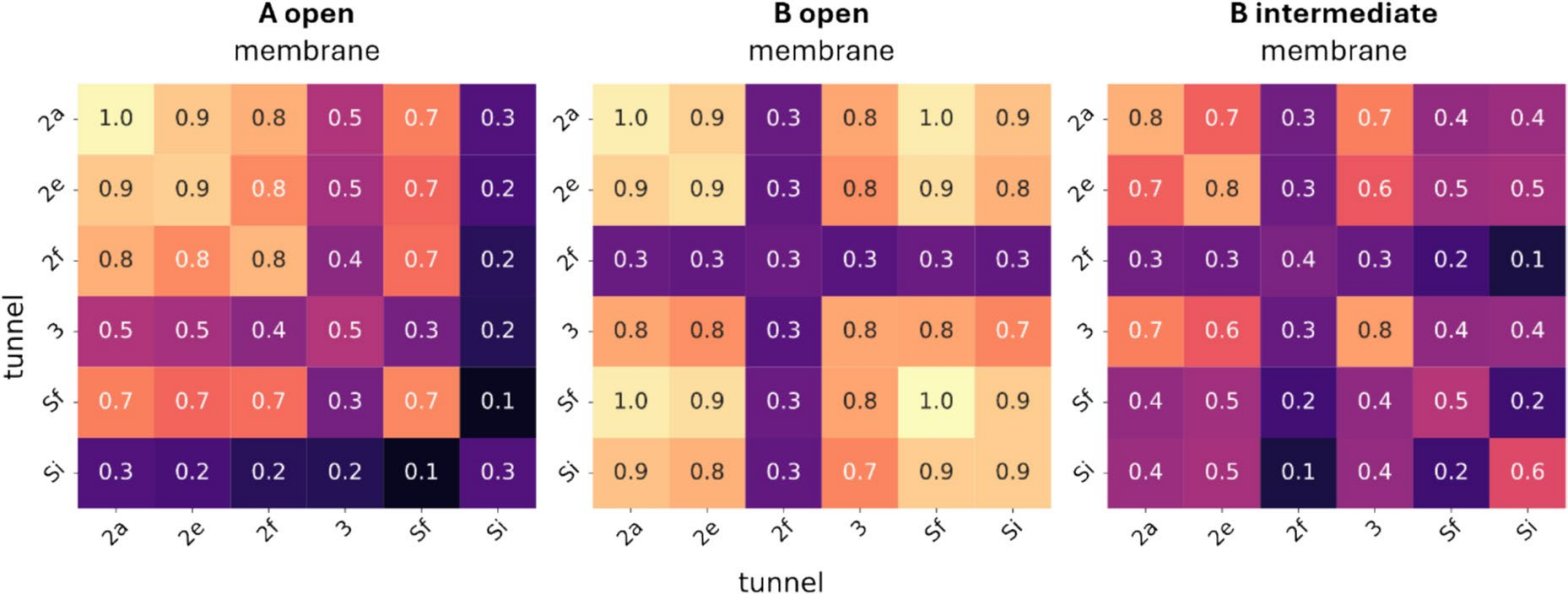
Relative time, in which a pair of tunnels opens together in the state trajectory. The numbers inside the cells mark the relative time, with maximum 1.0 and minimum 0.0. The colour scale is according to the relative time

For each pair of tunnels, we calculated the relative simulation time during which both tunnels are open simultaneously, closed simultaneously, or exhibit opposite behaviours. The relative time that two tunnels remain open together depends on the tunnel that is predominantly closed during the simulation. If this predominantly closed tunnel is open, the other tunnel tends to be open most of the time as well. Interestingly, even tunnels 2f and 3, which display opposing behaviours within and between the states, can be open simultaneously: tunnel 2f is open 31% of the time in state B_open, and for 27% of the simulation time in state B_open, both tunnels 2f and 3 are open together, see Fig. 6 and in online resource Fig. 8.

## Discussion

This study investigated the dynamic behaviour and conformational ensemble of CYP3A4 based on molecular dynamics (MD) simulations in a membrane environment and derived three distinct conformational states. In the following, we will present and explain our three main findings.

But before we start discussing those findings, we will validate our modelled systems and our assumption to cover the known conformational space by our starting structures. The PCA on the cartesian coordinates of the FG region is justified after analysing the tunnel exits, which are surrounded by the FG region, or by Paco et al. hydrogen deuterium exchange mass spectrometry study, identifying the FF′ region as a very flexible region [14]. After selecting the structures with maximal distance in the given PCA space, it is not surprising that 3NXU and 2V0M show strong deviation from 1TQN in the FF′ loop. Especially F213 and F215 adopt different conformations, which will be further analysed. Our modelling of the transmembrane helix is validated by high lDDT values, as presented in Mariani et al. study [71]. Moreover, the membranes integrity during the simulation is given, as the surface area per lipid is constant. Our average area per lipid value of 68.8 Å^2^ is in line with the literature: CHARMM-GUI gives a reference value of 68.3 Å^2^ for a POPC membrane [72]. We can also rely on our enzyme orientation in the membrane during the simulation. Although the tilt angle is high in the modelled membrane-enzyme complex, it relaxes to lower values during the simulation with an average of 59.9°, which fits well with the values in literature: in linear dichroism experiments in a POPC nanodisc the average tilt angle of 11 replica was 59.7° + − 4.1° [73], during a simulation of CYP3A4 in a DOPC membrane an average tilt angle of 56° + − 5° was estimated [22]. The same study [22] calculated the distance of the tunnel openings to the lipid bilayer center as well. Their results are in accordance with ours, tunnel 2f and 4 (in our study tunnel 3) are closer to the membrane compared to tunnel 2e and the solvent tunnels. Our membrane system is a simplified lipid bilayer, which does not represent the actual natural CYP environment. Although we are neglecting cholesterol effects [74], like thickening and ordering of the membrane, or charge effects, resulting in changes of the immersion depth [75], we see already influences of the membrane on the conformations of the CYP, which will probably increase with more complex membrane systems. Some of the parameters used to calculate the tunnels are different from parameters in other studies. The probe sphere is often set to 1.4 Å, which reflects the van der Waal radius of a water molecule [28, 29]. However, a recent study pointed out that water can pass through tunnels which are narrower than 1.4 Å [27].

With respect to our first finding, we want to stress the necessity of long simulations. The importance of simulations in the microsecond range is evident from our analysis of the bottleneck radii distributions of tunnel 3 compared to the radii in the crystal structures and simulations in literature. Tunnel 3, initially closed in crystal structures, opens significantly during simulations starting from these structures, as seen in state B_open. A study of Benkaidali [76], which focused on crystal data, detected solely four tunnels, instead of six in our study. Moreover, other studies [22, 73] have conducted simulations of CYP3A4 for 50–100 ns with and without a membrane. Baylon et al. [73], who simulated for 50 ns, claimed that tunnel 3 only opens upon insertion in a membrane, while Berka et al. [22], who simulated for 100 ns, observed an open tunnel 4 in systems with and without a membrane. We treat tunnel 4 and 3 in our study equally. Although both authors started their simulations from 1TQN, they obtained diverging results. The study with 100 ns simulations described in both systems, with and without membrane, open tunnels 3/4, while shorter simulations of 50 ns missed the open tunnel 3/4. Longer simulations, like our study with 1000 ns for each starting structure, sample the range of possible bottleneck radii more thoroughly, as our study reveals larger bottleneck radii for tunnel 3 compared to the aforementioned studies.

Moreover, the need for long simulations, even longer than our simulations, becomes apparent by looking at our non-converged simulations. The distinct conformational states of CYP3A4 are separated from each other by large energy barriers. We already span a broad conformational space of CYP3A4 by choosing five starting conformations, but our simulations are heavily depending on the starting conformations. Nevertheless, they indicate that state transitions are infrequent but possible, particularly in membrane-free systems known for their lower rigidity. Those rare state transitions suggest that complete conformational state changes are in general feasible. However, in our case in both systems the state transitions in between the PCCA + states were not sufficient to construct a valid MSM. Therefore, we do not discuss mean first passage time or populations sizes. In a previous study [18] the transition of an open to closed state and vice versa of P450cam was sampled in a 84 µs adaptive sampling simulation. Such simulations could help to elucidate the mechanism of state transitions after constructing a valid MSM.

Since our simulations depend strongly on their starting conformations, our findings align closely to the state definitions of Benkaidali et al. [20] based on open tunnels in crystal structures and size and physicochemical properties of the crystalized ligands. They proposed three states: O2 (open), O1 (open) and C (closed), corresponding to the crystal structures 2V0m (O2), 3NXU (O1), and 1TQN and 6BD6 (C). Therefore, O2 is our state A_open, O1 is B_open and C is B_intermediate, as our states originate from the same crystal structures. In both Benkaidali’s state O2 and our state A_open, tunnels 2f, 2a, and at least one of the solvent tunnels are widely open. In contrast, state C/B_intermediate has a smaller binding site, and in state O1/B_open, only tunnel 2a remains open according to Benkaidali. However, our results indicate that tunnel 3 and tunnel Sf are open in state B_open as well. Tunnel 3 is generally overlooked in the literature’s state definitions.

Interestingly, our results are not in line with the state definition of Yu et al. [19], who classified 2V0M and 3NXU as closed state, while they are part of state A_open and B_open in our study, respectively. The key difference between Benkaidalis and Yus approaches lies in their characterization criteria: Benkaidali et al. considered ligand properties, such as hydrophobicity, hydrophilicity, ligand size, and ligand position within the binding site, while Yu et al. focused solely on tunnel configurations in the crystal structures. By including the ligand properties, they accounted indirectly for the flexibility which is necessary to let a ligand pass through a tunnel. Hence, even if the tunnel is closed in the crystal structure, the ligand must have found its way inside the CYP beforehand, and the tunnel is recognized by Benkaidalis method. We simulated this dynamic behaviour directly. Moreover, we can elucidate the opening and closing mechanisms by the interaction patterns, as we did for tunnel 2f and 3. Therefore, our second finding is, that it is crucial to take the dynamics of a state into account and not only a single static structure to understand the behaviour and characteristics of a state.

The systems without membrane cannot be easily categorized into those predefined states: open or closed/ intermediate. The openness of the tunnels differs in between the states originating in the same crystal structures between the systems with and without membrane: simulations starting from 6BD6 developed to a state with more open tunnels (state 2) in systems without a membrane, while the resulting state in systems with membrane is the more closed state B intermediate. Since the backbone conformation in comparable states, underlying simulations started from the same starting structure, stays similar between the systems with and without membrane, indicated by shared minima in the tICA space, the membrane seems to influence the orientation of the residues and their interactions. Hence, another valuable aspect in our study is the inclusion of the membrane in the simulations and the following state definition. Former studies have already demonstrated the membrane’s influence on the tunnel opening. For instance, in CYP17A1 the openings of tunnels 2fg, which passes through an FG loop, and 2f are favoured during simulations in the membrane [77]. The authors found a mechanism in which the membrane interacts with an internal aromatic gate and tunnel 2fg opens consequently. Similarly, in CYP2C9 the membrane facilitates the opening of tunnel 2a by opening an internal aromatic gate [24]. Other authors observed similar stabilizations in aromatases [78]. Our study confirms that the membrane significantly alters the bottleneck radii distributions for all tunnels, particularly those near the F helix, G helix, or their connecting loops (e.g., tunnels 2a, 2f, 3, Si, Sf, and 2c). One possible reason for this observation is that the membrane rigidifies the CYPs. Hydrogen Deuterium exchange mass spectrometry (HDX-MS) experiments of CYP3A4 either in emulgent or in a nanodisc showed impressively that especially the FG region is affected by membrane rigidification effects. Our results indicate the biggest flexibility changes in the FF′ loop, measured by dihedral entropy. This observation is supported by relative membrane interaction times, which reveal significant differences in membrane interactions with the FF′ loop compared to the G and F helices: while ARG212, PHE213, ASP 214, PHE215 and LEU216 in the FF’ loop in state A_open interact with the membrane the entire time of the simulation, they show varying relative interaction times in the states of group B. Moreover, the rigidification is also an explanation for the less frequent state transitions in the system with membrane compared to the systems without membrane (Online resource Figs. 1 and 3).

Despite the broad impact of the membrane on tunnel dynamics, we focused on tunnel 3 and 2f, as they show extreme changes in between the states and the systems with and without membrane. Tunnel 2f is widest open in state A_open in the membrane system and generally closed by hydrophobic interactions in between the FF′ loop and the A helix. We identified a mechanism which stabilizes the open conformation of tunnel 2f in state A_open. In this state the membrane interacts with the complete FF′ loop (ARG 212, PHE213, ASP214, PHE215, LEU216) in contrast to the other two states. This prevents the phenylalanine in the FF′ loop (PHE215) from interacting with the phenylalanine in the A helices (PHE57). In 2V0M, which is the base of state A_open, PHE215 already adopts a distinct conformation, particularly when compared to the crystal structures underlying state B_intermediate. Additionally, lipids which are entering tunnel 2f in its open form, stabilize the tunnel even further, a phenomenon already described by other authors [24, 79]. Previous studies [73] proposed that the membrane facilitates the opening of the aromatic gate, composed of phenylalanines 213, 215, 241, 220, 108 and 304. In our study we found that tunnel 3, which is directly facing the membrane, is mainly closed by the aromatic phenylalanine 213 interacting with other residues of the aromatic gate. Therefore, we expected tunnel 3 to be wider open in the systems with membrane, because of expected aromatic gate opening effects. However, we found the widest bottleneck for tunnel 3 in the system without membrane, namely in state 0. Interestingly, state A_open in systems with membrane, in which tunnel 3 is closed, and state 0 in systems without membrane, exhibiting widely open tunnel 3 s, generated from the same starting conformations. The discrepancy between state A_open and state 0 may be due to the conformation getting trapped in a closed form and the membrane’s rigidifying effect hindering conformational changes. Alternatively, the membrane might interact with residues indirectly affecting tunnel 3’s opening and closing. Therefore, our third finding is, that CYP3A4 must be simulated in a membrane, since the membrane has great influence on the CYPs dynamics and the interaction network.

The tunnels of CYP 3A4 codetermine the specificity of the enzyme. The tunnels do not only vary in their bottleneck radii but also in their physicochemical properties of the bottleneck residues lining the tunnels. Tunnel 3 and tunnel 2f are the most hydrophobic tunnels, while tunnel 2a is not only lined by hydrophobic residues but also by hydrophilic residues (Table 1) and is less hydrophobic. The most hydrophilic tunnels are the solvent tunnels Sf and Si. However, tunnel 2e, which is not facing the membrane either, is less hydrophilic compared to the solvent tunnels. Therefore, it can be a possible exit pathway for less hydrophilic metabolites. From literature it is known that hydrophobic ligands like testosterone [25] or progesterone [80] enter via tunnel 3, and that caffeine [12] is also accumulating close to tunnel 3, 2a and 2f. This corresponds well with the hydrophobic nature of the bottleneck residues of those three tunnels which are also facing the hydrophobic membrane. The hydrophobic character of tunnel 2f is further emphasized by the entering of a lipid into the tunnel. The more hydrophilic tunnel 2e is described in literature as a secondary egress tunnel [8] and tunnel S is called the solvent tunnel which can also serve as an exit tunnel, i.e. for caffeine metabolites [13], which are more hydrophilic than caffeine itself. Our docking mainly distinguishes the tunnels by their bottleneck radii and the size of the ligands: caffeine (smaller) shows lower energy barriers than progesterone (bigger). Progesterone and caffeine have higher energy barriers in state B_intermediate in tunnels 2a, Sf and 3, which is in line with our original state definition, which describes state B_intermediate to be more closed than the other two states. In six cases, tunnel Si in state A_open and B_open and tunnel 3 in state B_intermediate, the bottleneck radius of the tunnel conformation is big enough for ligand passage, but the energy profiles show high barriers. In these states and tunnels the lining residues can have changed or moved, and other functional groups point into the tunnel, and the physicochemical properties of the residues along the tunnel influence the ligand passage. Moreover, both ligands can exit not only through at least one conformation of the hydrophobic tunnels 2a, 2f and 3 but also through at least one conformation of the solvent facing tunnels 2e and Si and Sf. In our simulations we generated an ensemble of tunnel conformations, and every ligand can exit via one of those conformations. This supports the conformational selection hypothesis.

Another question which is often raised is if the tunnel opening and closing is correlated. Tunnel 2e, 2a and at least one tunnel of the solvent tunnels Sf and Si are open in all states more than 60% of the time and are not influenced by the opening and closing of the other tunnels. However, tunnel 2f and 3 exhibit opposite behaviours across states: in states in which tunnel 3 is open, tunnel 2f is mainly closed and vice versa. Our correlation analysis showed that they still open together, always limited by the more closed tunnel in the state. This is supported by the interaction analysis. Tunnel 2f is closed by PHE215 or LEU216, both in the FF′ loops, interacting with PHE57, in the A helix, while tunnel 3 is closed by PHE213. The closing residues are distinct for every tunnel and are not interacting with each other. Therefore, the tunnel opening and closing is not correlated.

## Conclusion

In our study we successfully captured CYP3A4’s promiscuity in a membrane environment by identifying three distinct states: A_open, B_open, and B_intermediate. We observed that the primary differences between groups A and B are associated with tunnels 2f and 3, with state B_intermediate being less accessible to larger substrates due to its smaller bottleneck radii. Importantly, we elucidated a mechanism where the membrane stabilizes the open conformation of tunnel 2f in state A_open. To our knowledge, we are the first study to describe the states of CYP3A4 dynamically and in a membrane environment. Although we employed a neutrally charged and simple membrane system, we were already able to identify membrane effects.

Our findings highlight on one hand the significance of microsecond-scale simulations, revealing that bottleneck radii in crystal structures differ from those observed in simulations. Moreover, we want to point out the need for even longer simulations than 1–2 µs to reach converged simulations, since CYP3A4 has a broad conformational space with high energy barriers in between the conformational states. And on the other we want to emphasize the importance of simulating CP3A4 in a membrane. Those insights are crucial for improving metabolization prediction models by incorporating various conformational states. With extended simulations, we anticipate being able to determine state probabilities more accurately. Additionally, the knowledge gained from this study can be applied to manipulate specific states to enhance or inhibit substrate metabolization, offering valuable implications for drug development and metabolic engineering. This work can guide new mutations in CYP3A4 to stabilize or destabilize a conformation. We identified critical state defining residues and their interactions. Moreover, we characterized the tunnel opening patterns per state, which in turn can help determine which state should be stabilized by mutations or inhibitors/substrates.

## Supplementary Information

Below is the link to the electronic supplementary material.

Supplementary file1 (PDF 4001 KB)

## Data Availability

The authors declare that the data supporting the findings of this study are available within the paper and its Supplementary Information files. Should any raw data files be needed in another format they are available from the corresponding author upon reasonable request.
