# Supplementary material for: From closed to open: three dynamic states of membrane-bound cytochrome P450 3A4
Source: J Comput Aided Mol Des. 2025 Mar 17;39(1):12. doi: 10.1007/s10822-025-00589-1 (PMC11913904; doi:10.1007/s10822-025-00589-1)
Supplement: Supplementary file 1 — Supplementary file1 (PDF 4001 KB) [file 10822_2025_589_MOESM1_ESM.pdf]

Supplementary Information (SI) for "**From closed to open: Three dynamic states of membrane-bound Cytochrome P450 3A4"**

Journal of computer-aided molecular design

Vera A. Spanke[1], Valentin J. Egger-Hoerschinger[1], Veronika Ruzsanyi[2], Klaus R. Liedl[1*]

[1]Department of Theoretical Chemistry, Universität Innsbruck, Innsbruck, Austria

[2]Department of Breath Research, Universität Innsbruck, Innsbruck, Austria

[*]corresponding author, email: Klaus.Liedl@uibk.ac.at

Table 1 Bottleneck residues for MSM construction:

With/ without membrane	A121, I120, D217, V376, M371, F241, T224, T309, R440, G109, N104, FE502, I230, L51, F219, P227, R105, F304, A370, F108, K115, P107, L211, P485, T207, HEME501, Y53, R372, S478, L210, G481, R106, Q78, M181, V111, V240, F57, E374, K173, L216, I50, F220, L483, Q79, I303, F213, D76, Q484, I369, H54, L221, G480, E122, V225, R212, S119, P242, Y307, K208, S312, A305, I223, Y25, L482, F215, D214, I300, L479, E308

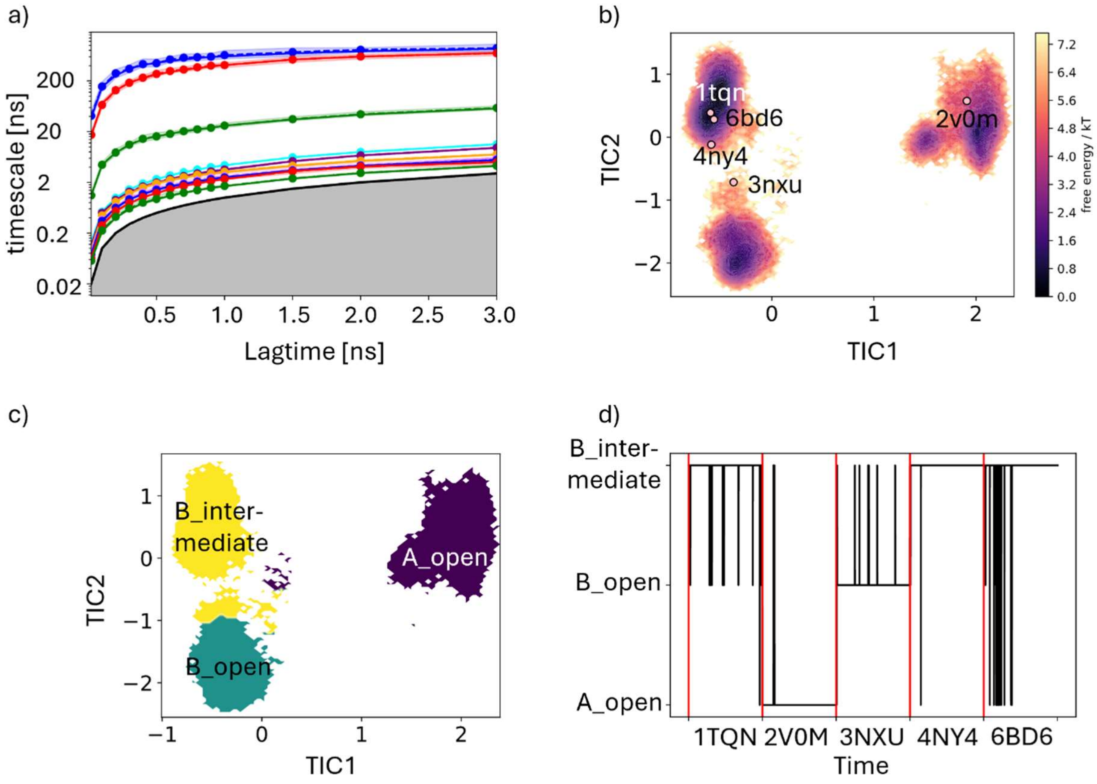

Fig 1 MSM construction for the systems with membrane. In a) the implied timescale plot illustrates how implied timescales vary with different lag times. The appropriate lag time for constructing an MSM is identified when the timescales reach a plateau, indicating that the function becomes constant. In b) is shown the tICA space of the combined simulations of the systems with membrane. The pink filled circles mark the starting conformations of the crystal structures. In c) is shown the tICA space coloured

according to the MSM state membership. And plot d) describes the state transitions observed in the original simulations, with the starting crystal structures plotted along the x-axis

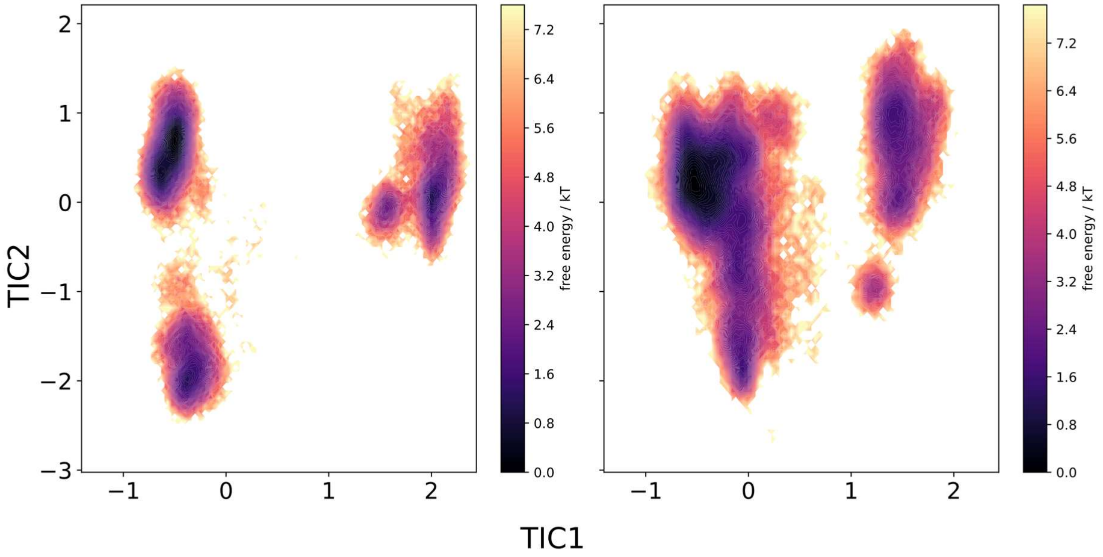

Fig. 2 tICA spaces of systems with membrane (left) and systems without membrane (right). They both share the same space and can be directly compared

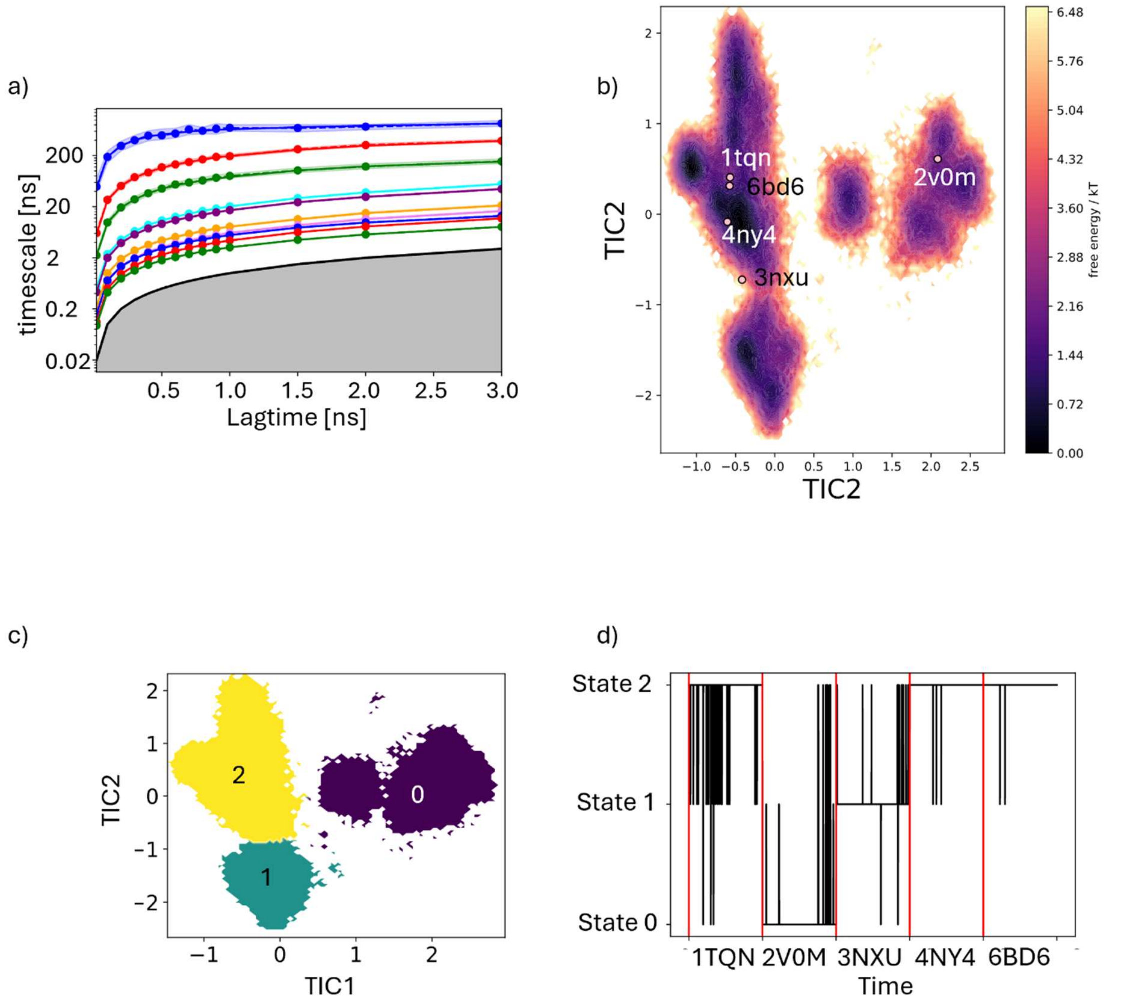

Fig. 3 MSM construction for the systems without membrane. In a) the implied timescale plot illustrates how implied timescales vary with different lag times. The appropriate lag time for constructing an MSM is identified when the timescales reach a plateau, indicating that the function becomes constant. In b) is shown the tICA space of the combined simulations of the systems without membrane. The pink filled circles mark the starting conformations of the crystal structures. In c) is shown the tICA space coloured according to the MSM state membership. And plot d) describes the state transitions observed in the original simulations, with the starting crystal structures plotted along the x-axis

Table 2 Bottleneck residues of contact analysis at the bottlenecks

With/ without membrane	Y25, G26, T27, H28, S29, H30, F33, G40, P41, P43, L44, F46, L47, G48, N49, I50, L51, S52, Y53, H54, K55, G56, F57, C58, M59, F60, D61, M62, E63, W72, F74, Y75, D76, G77, Q78, Q79, P80, V81, L82, A83, I84, V93, L94, E97, C98, Y99, S100, V101, F102, T103, N104, R105, R106, P107, F108, G109, P110, V111, G112, F113, M114, K115, S116, A117, I118, S119, I120, A121, E122, D123, E124, E125, W126, K127, R128, L129, R130, S131, L133, T136, F137, T171, L172, K173, D174, V175, F176, G177, A178, Y179, S180, M181, V183, I184, T185, S186, S188, F189, I193, S195, P202, F203, V204, E205, N206, T207, K208, K209, L210, L211, R212, F213, D214, F215, L216, D217, P218, F219, F220, L221, S222, I223, T224, V225, F226, P227, F228, L229, I230, P231, I232, L233, E234, L236, N237, I238, C239, V240, F241, P242, R243, E244, V245, T246, N247, F248, L249, R250, K251, S252, V253, K254, R255, M256, F271, L272, D292, L293, E294, V296, A297, Q298, S299, I300, I301, F302, I303, F304, A305, G306, Y307, E308, T309, T310, S311, S312, V313, L314, S315, F316, I317, M318, Y319, E320, L321, V360, T363, L364, L366, F367, P368, I369, A370, M371, R372, L373, E374, R375, V376, C377, K378, G391, V392, V393, V394, M395, I396, P397, S398, Y399, A400, T433, P434, F435, G436, S437, G438, P439, R440, N441, C442, I443, F447, A448, N451, M452, Q472, L475, K476, L477, S478, L479, G480, G481, L482, L483, Q484, P485, E486, K487, P488, V489, HEME501

Table 3 State populations in percent of the MSM, for systems with membrane and without membrane

With membrane		Without membrane	
A_open	20%	0	20%
B_open	20%	1	20%
B_intermediate	60%	2	60%

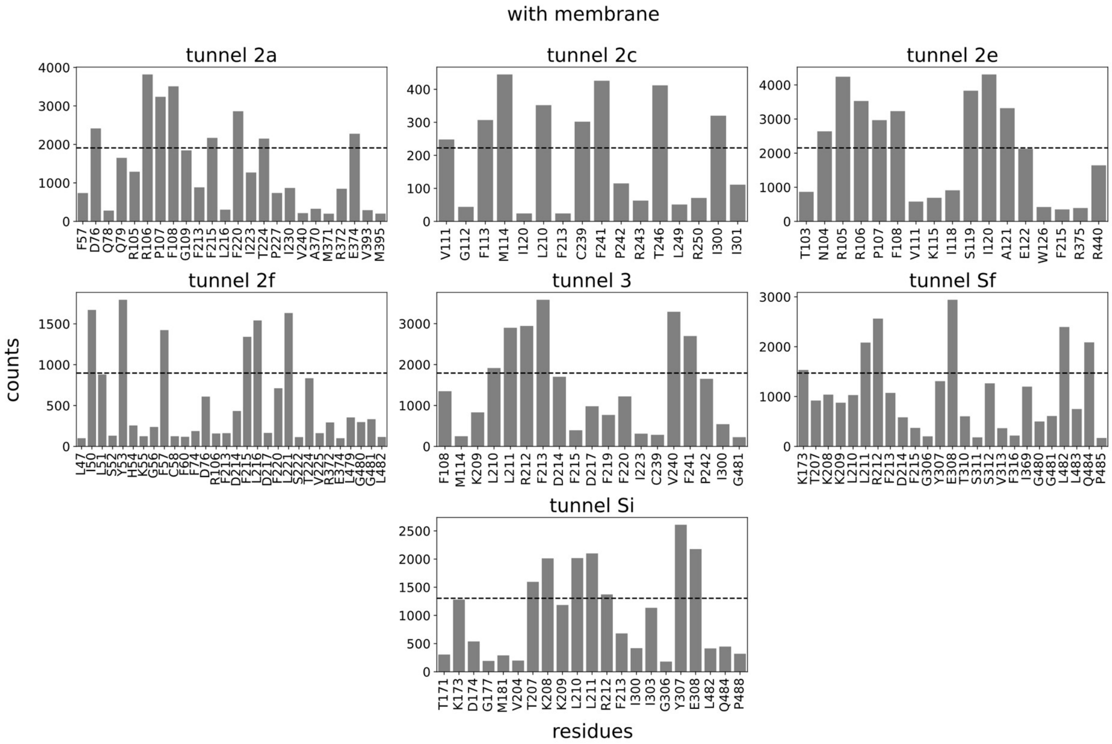

Fig. 4 Bottleneck residues in systems with membrane for each tunnel. The hight of the bar represents how often this residue is a bottleneck residue for the respective tunnel across the combined trajectory of all five simulations. The dashed lines mark 50% of the counts of the residue with the maximum counts

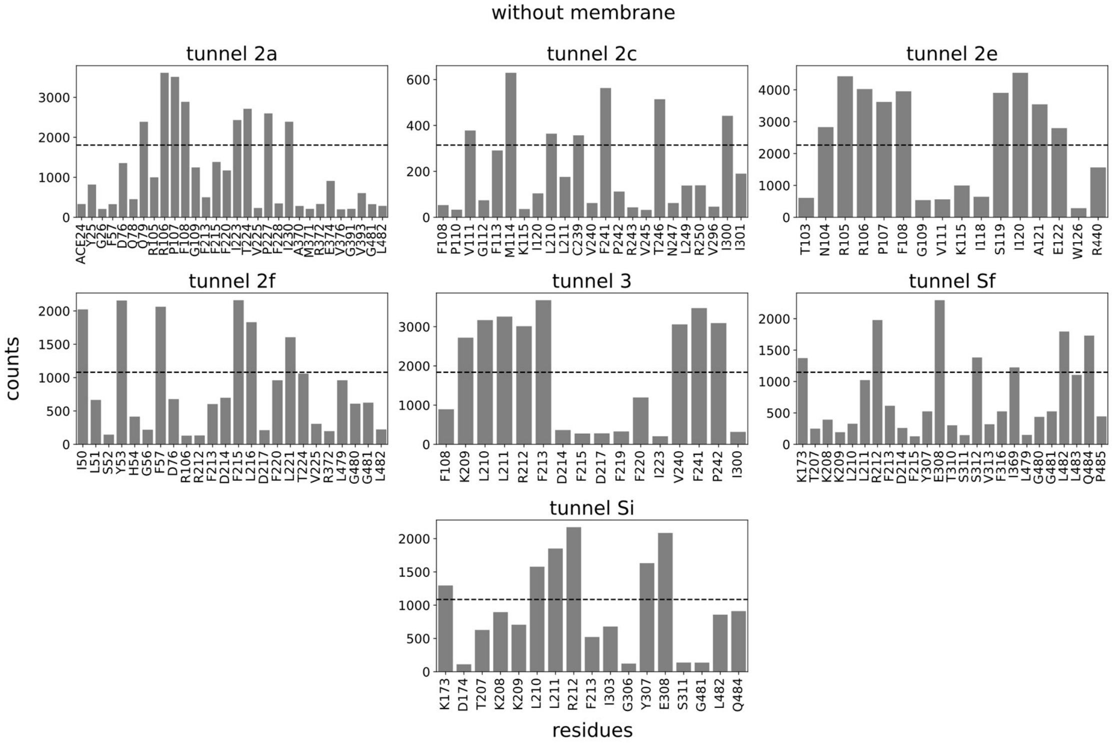

Fig. 5 Bottleneck residues in systems without membrane for each tunnel. The hight of the bar represents how often this residue is a bottleneck residue for the respective tunnel across the combined trajectory of all five simulations. The dashed lines mark 50% of the counts of the residue with the maximum counts

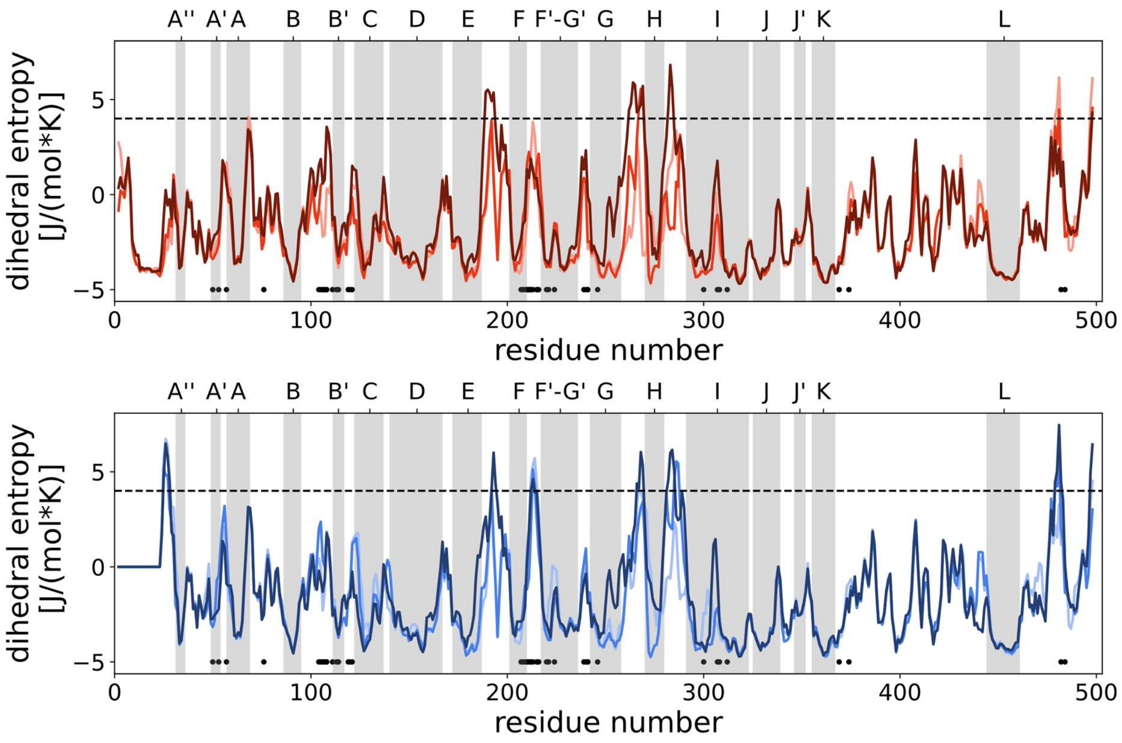

Fig. 6 The dihedral entropies for different states are shown with colour shading. The states for systems with a membrane are depicted in shades of red: light red for state A_open, red for state B_open, and dark red for state B_intermediate. For systems without a membrane, the dihedral entropies are represented in shades of blue: light blue for state 0, blue for state 1, and dark blue for state 2. The grey shaded areas indicate secondary structure elements, which are displayed above the plots. Black circles mark residues that appear as bottleneck residues more than 50% of the time relative to the most frequent bottleneck residue

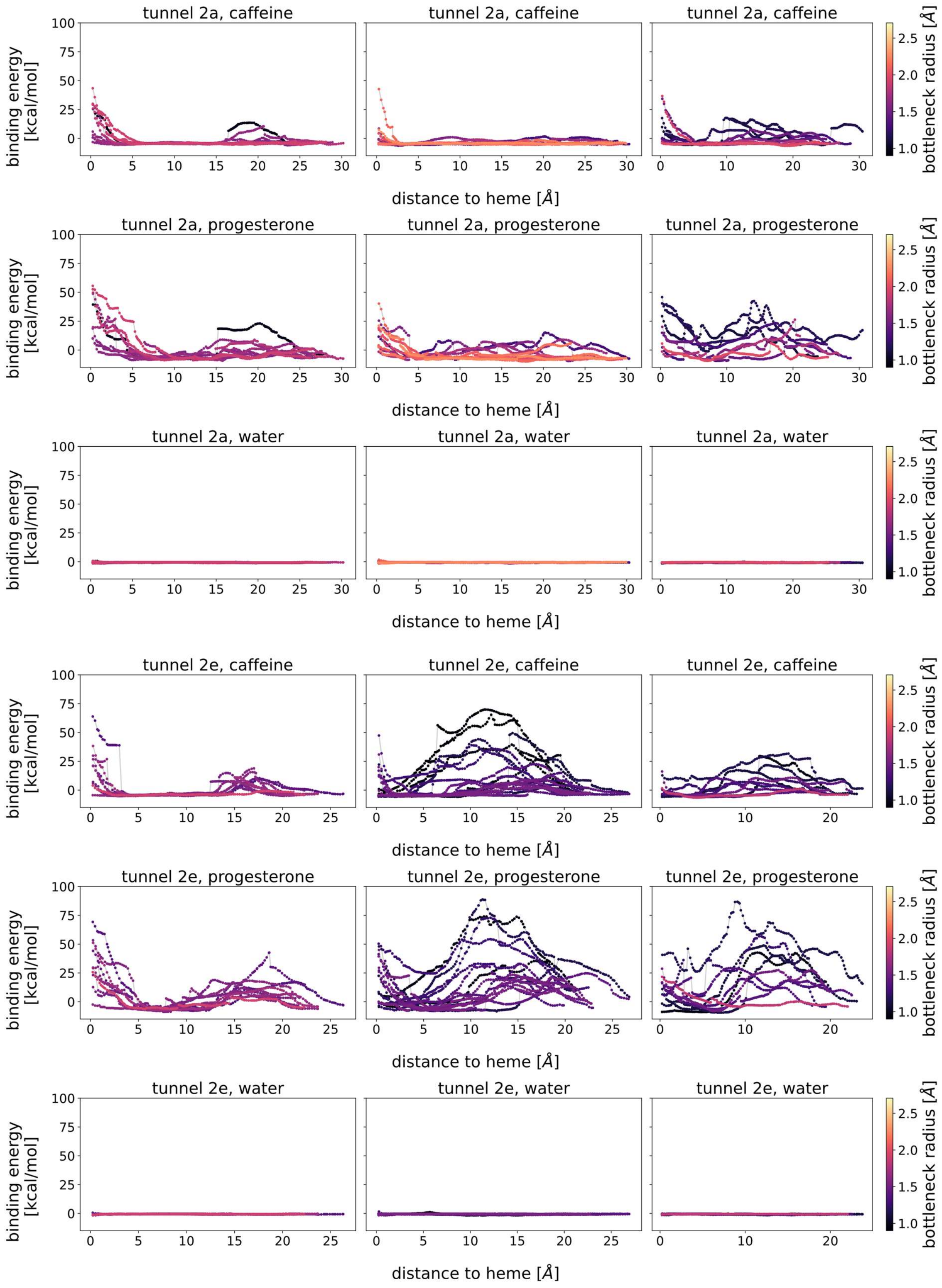
tunnel 2a, caffeine
tunnel 2a, progesterone
tunnel 2a, water
tunnel 2e, caffeine
tunnel 2e, progesterone
tunnel 2e, water
binding energy [kcal/mol]
distance to heme [Å]
bottleneck radius [Å]

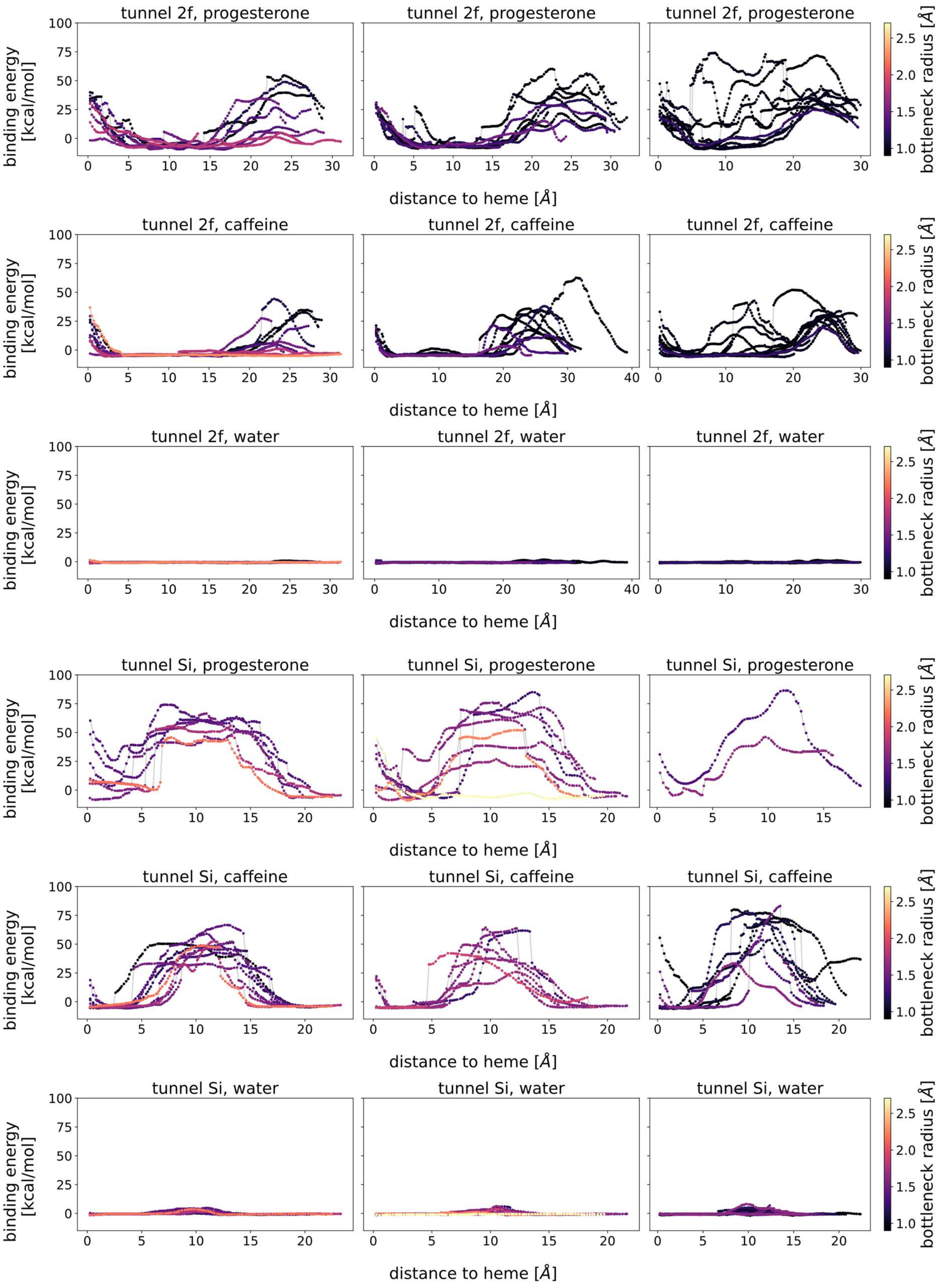
tunnel 2f, progesterone
tunnel 2f, progesterone
tunnel 2f, progesterone
tunnel 2f, caffeine
tunnel 2f, caffeine
tunnel 2f, caffeine
tunnel 2f, water
tunnel 2f, water
tunnel 2f, water
tunnel Si, progesterone
tunnel Si, progesterone
tunnel Si, progesterone
tunnel Si, caffeine
tunnel Si, caffeine
tunnel Si, caffeine
tunnel Si, water
tunnel Si, water
tunnel Si, water
binding energy [kcal/mol]
distance to heme [Å]
bottleneck radius [Å]
100
75
50
25
0
0
5
10
15
20
25
30
40
1.0
1.5
2.0
2.5

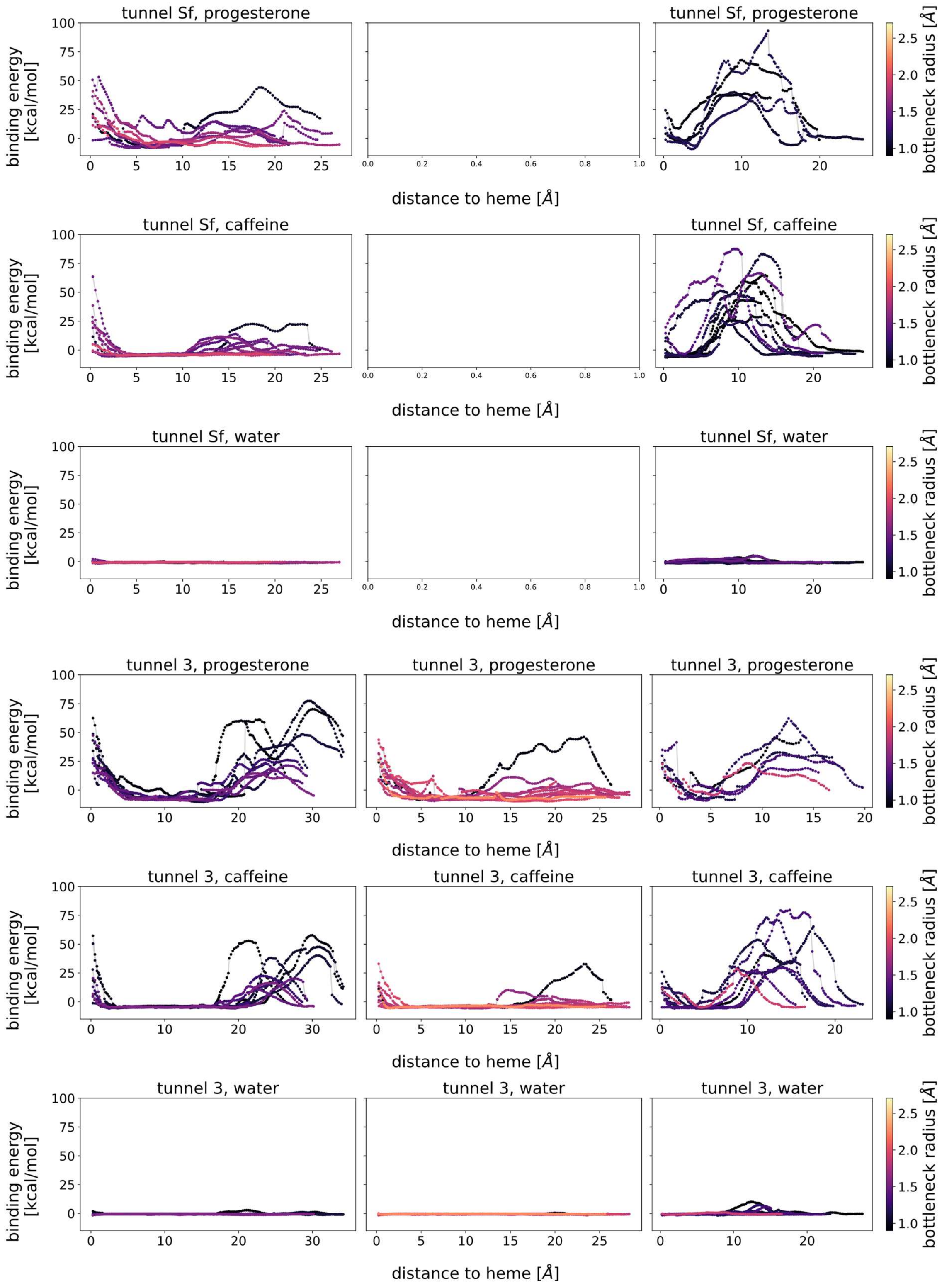

Fig. 7 Energy profiles of caffeine, progesterone and water for each tunnel in state A_open, B_open and B_intermediate. For each state, the ligands were docked into the same tunnels. Dark purple lines represent energy profiles for tunnels with small bottleneck radii, while light orange lines belong to tunnels with large bottleneck radii

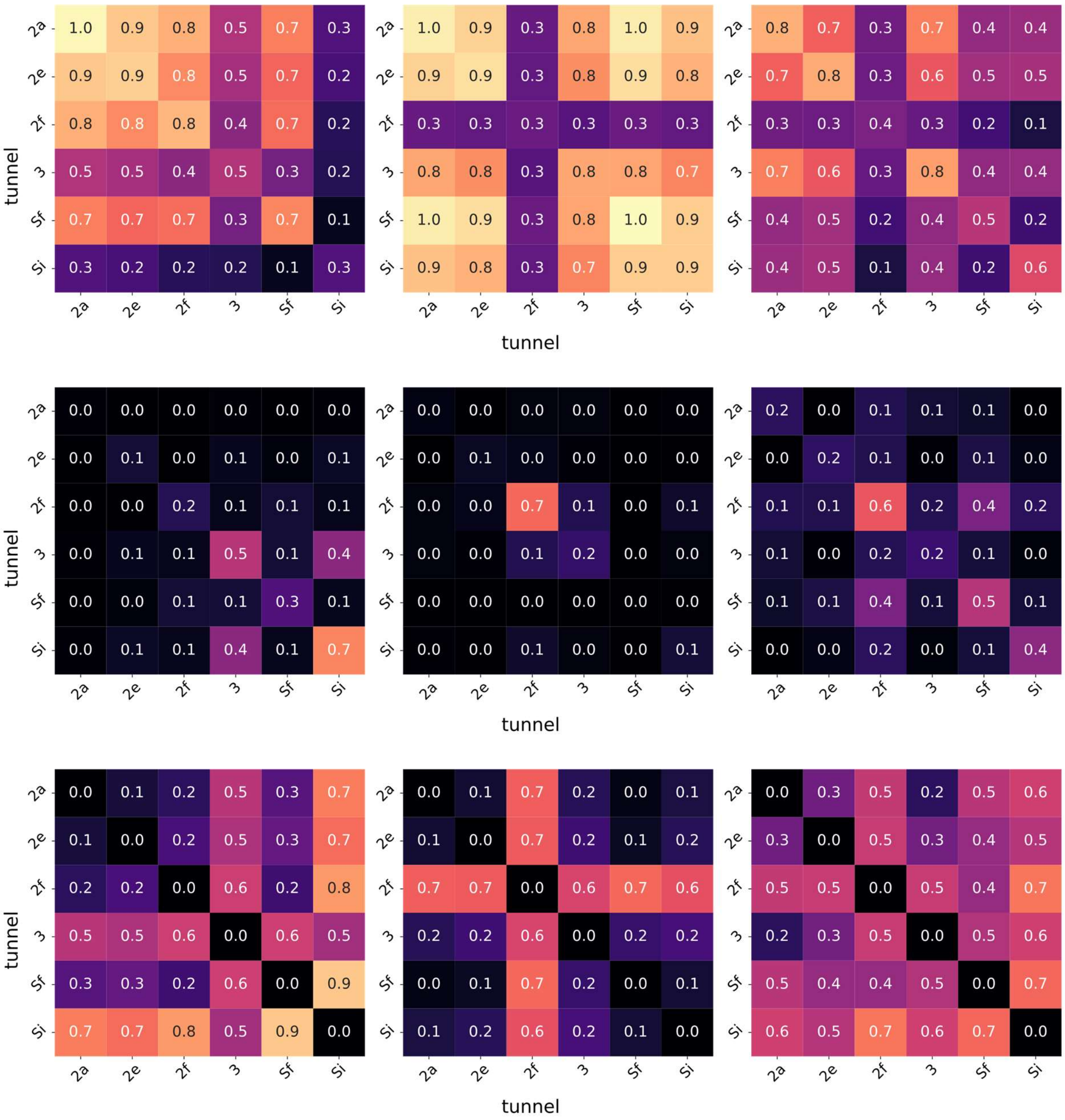

Fig. 8 Relative time in systems with membrane, in which a pair of tunnels opens together (top row), closes together (middle row), or is behaving oppositely (bottom row) in the state trajectory. The numbers inside the cells mark the relative time, with maximum 1.0 and minimum 0.0. The colour scale is according to the relative time

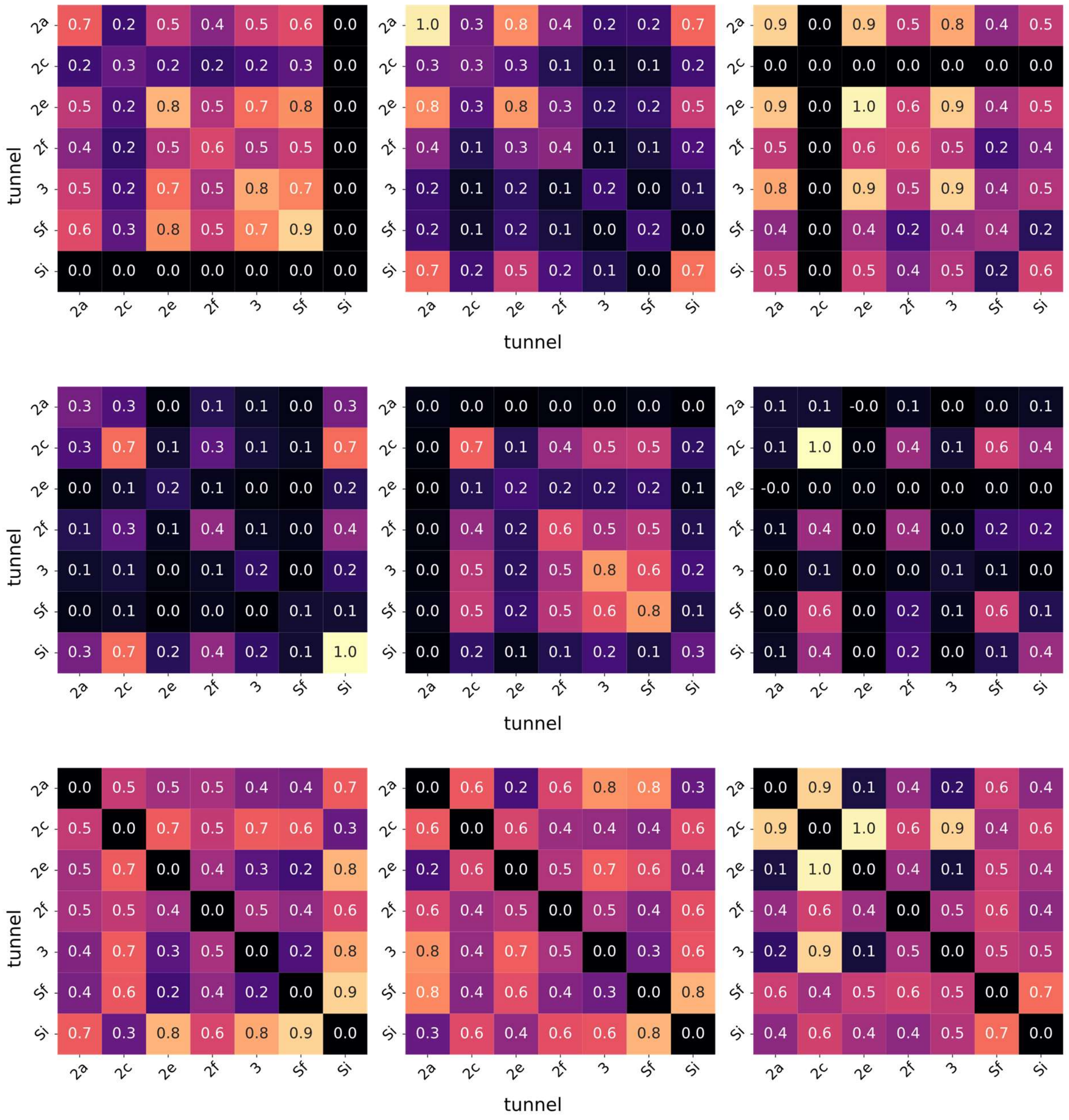

Fig. 9 Relative time in systems without membrane, in which a pair of tunnels opens together (top row), closes together (middle row), or is behaving oppositely (bottom row) in the state trajectory. The numbers inside the cells mark the relative time, with maximum 1.0 and minimum 0.0. The colour scale is according to the relative time

Table 4 modelled residues for each starting structure

PDB code	Transmembrane helix (AlphaFold2)	GH loop (MOE)	HI loop (MOE)
1TQN	M1, A2, L3, I4, P5, D6, L7, A8, M9, E10, T11, W12, L13, L14, L15, A16, V17, F18, L19, V20, L21, L22, Y23, L24, Y25, G26, T27, H28, S29, H30		N280, S281, K282, E283, T284, E285, S286, H287, K288, A289
2V0M	M1, A2, L3, I4, P5, D6, L7, A8, M9, E10, T11, W12, L13, L14, L15, A16, V17, F18, L19, V20, L21, L22, Y23, L24, Y25, G26, T27, H28, S29, H30	D263, T264, Q265, K266, H267, R268, V269, D270	S278, Q279, N280, S281, K282, E283, T284, E285, S286, H287, K288, A289, L290, S291, D292
3NXU	M1, A2, L3, I4, P5, D6, L7, A8, M9, E10, T11, W12, L13, L14, L15, A16, V17, F18, L19, V20, L21, L22, Y23, L24, Y25, G26, T27, H28, S29, H30	T264, Q265, K266, H267, R268	N280, S281, K282, E283, T284, E285, S286, H287, K288, A289, L290
4NY4	M1, A2, L3, I4, P5, D6, L7, A8, M9, E10, T11, W12, L13, L14, L15, A16, V17, F18, L19, V20, L21, L22, Y23, L24, Y25, G26, T27, H28, S29, H30	T264, Q265, K266, H267, R268, V269, D270, F271	Q279, N280, S281, K282, E283, T284, E285, S286
6BD6	M1, A2, L3, I4, P5, D6, L7, A8, M9, E10, T11, W12, L13, L14, L15, A16, V17, F18, L19, V20, L21, L22, Y23, L24, Y25, G26, T27, H28, S29, H30	E262, D263, T264, Q265, K266, H267, R268, V269, D270	N280, S281, K282, E283, T284, E285, S286, H287, K288

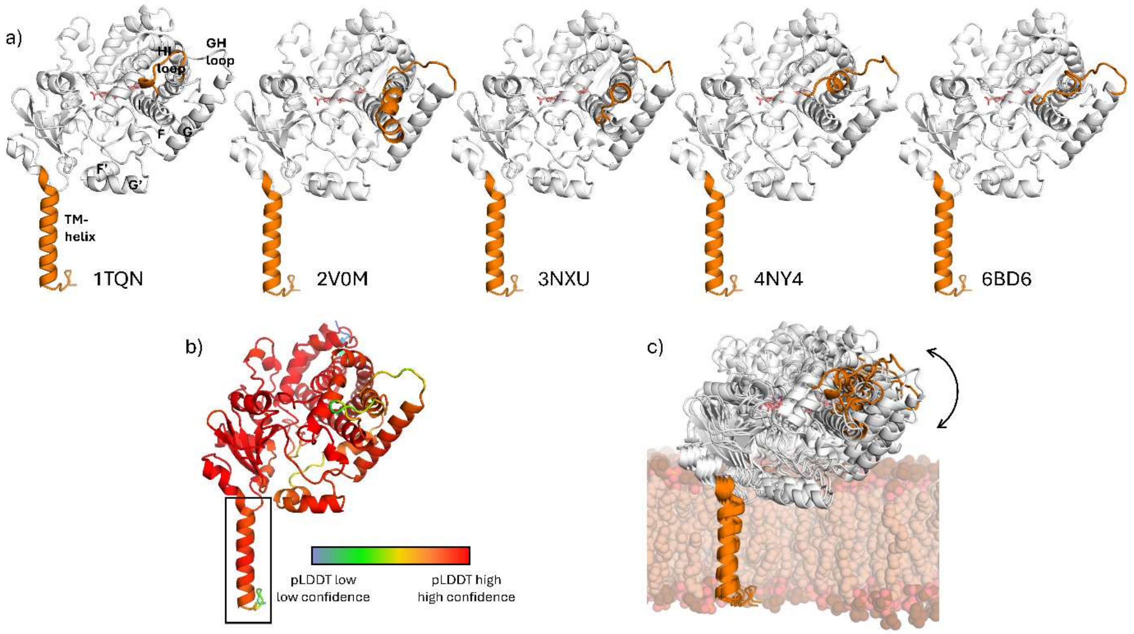

Fig. 10 Overview of the modelled regions. a) structure of the 5 selected starting pdb structures, labelled with the pdb code. In orange cartoon representations are the modelled regions highlighted, which are missing in the original pdb structures. b) The by AlphaFold2 predicted structure coloured according to the pLDDT values given by AlphaFold2, ranging from blue coloured, low confidence values to red coloured, high confidence values. c) all starting structures, consisting of the 5 pdb structures with their modelled regions, aligned to 1TQN. The arrow indicated the changing tilt angle

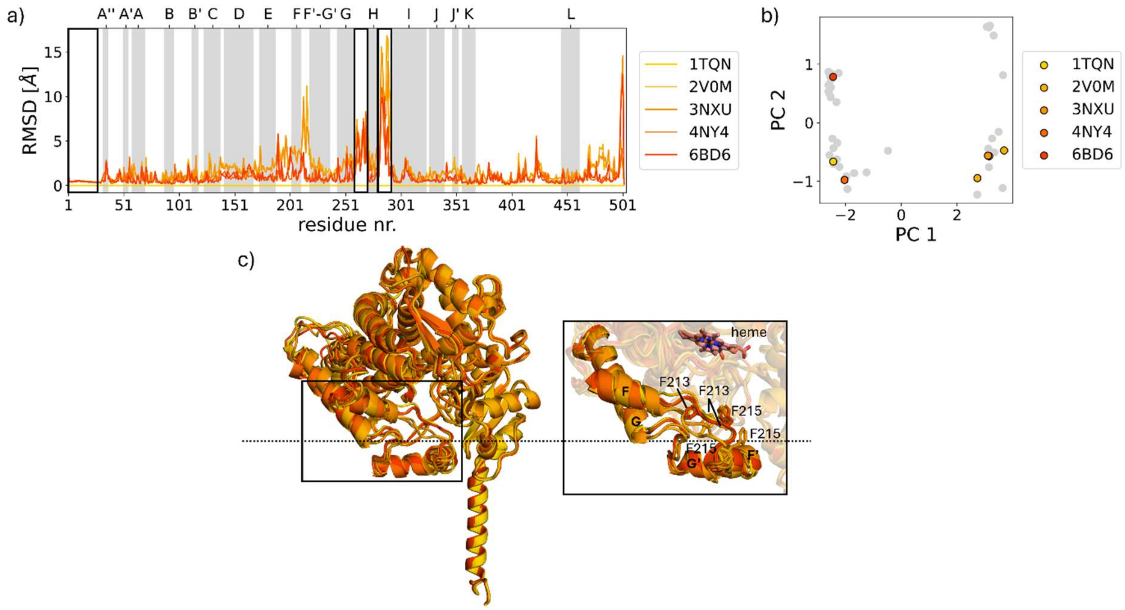

Fig. 11 Comparison of the 5 starting structures. a) Root mean square deviation for each residue in the five starting structures after alignment to 1TQN. Each line represents another starting structure. The grey shaded background marks secondary structure elements, which are labelled on top. The black squared regions are modelled regions. b) The principal component analysis space of the FG region of the 39 pdb structures. In grey are pdb structures, which we did not select for further simulations, in orange shaded colours are our selected pdb structures for further simulations. c) Alignment of the starting structures. Different colours represent each starting structure with the same colour coding as in 11a)b). The dashed line indicates the membrane, the part beneath the line is inserted into the membrane. The part surrounded by the black square is depicted again in more detail. F213, F215 and the heme including the iron are shown in stick representation

Table 5 PDB codes. In the first row are all PDB codes listed, which have the structure of CYP3A4. In the second row are only the PDB codes with a CYP3A4 in which the FG region is completely resolved

PDBs with CYP 3A4	4D7D, 6UNE, 6UNK, 2V0M, 6UNL, 5TE8, 7UFA, 4D78, 6O09, 7KVM, 7KVJ, 6DAB, 6DAL, 4I3Q, 6BDI, 6BD5, 6OOB, 4K9T, 4I4H, 7KVQ, 1W0G, 7KS8, 3UA1, 4NY4, 6UNM, 5A1R, 3TJS, 6UNJ, 7UAY, 6DA8, 4K9U, 7KVP, 1W0F, 4I4G, 7LXL, 5G5J, 6BDH, 6DAJ, 6DAC, 4K9V, 1W0E, 6DA5, 6BD7, 4K9X, 6DA2, 6BDK, 2J0D, 7KVO, 6DAG, 5VCC, 7KVH, 7UFD, 7UFC, 6UNI, 6UNG, 7UAZ, 7KVI, 3NXU, 6DAA, 7KVN, 6MA8, 6MA6, 6BDM, 6DA3, 6BCZ, 5VC0, 6BD6, 6BD8, 6OOA, 4K9W, 7KSA, 1TQN, 5A1P, 7UF9, 6UNH, 7UFB
PDBs with CYP3A4 and FG region	6BDI, 1W0E, 4K9V, 6OOB, 7KVQ, 6MA8, 6MA6, 6BD6, 5VC0, 6BD8, 4K9W, 4D78, 5A1P, 6BD7, 7UFD, 2V0M, 7UFC, 4D7D, 4I3Q, 3UA1, 4NY4, 7KVO, 5VCC, 7KVH, 1W0G, 4I4H, 4K9T, 6BDK, 1TQN, 6O09, 6BD5, 7UFB, 5A1R, 4I4G, 3NXU, 1W0F, 7KVN, 6BCZ, 5G5J

Table 6 tilt angles of membrane ensembles prior and during MD simulations

PDB code	1TQN	2V0M	3NXU	4NY4	6BD6
Tilt angle prior [°]	75.3	74.9	70.8	74.4	75.9
Tilt angle average during [°]	59.8 ± 7.2	56.8 ± 8.7	64.1 ± 7.6	57.2 ± 6.1	61.4 ± 7.5

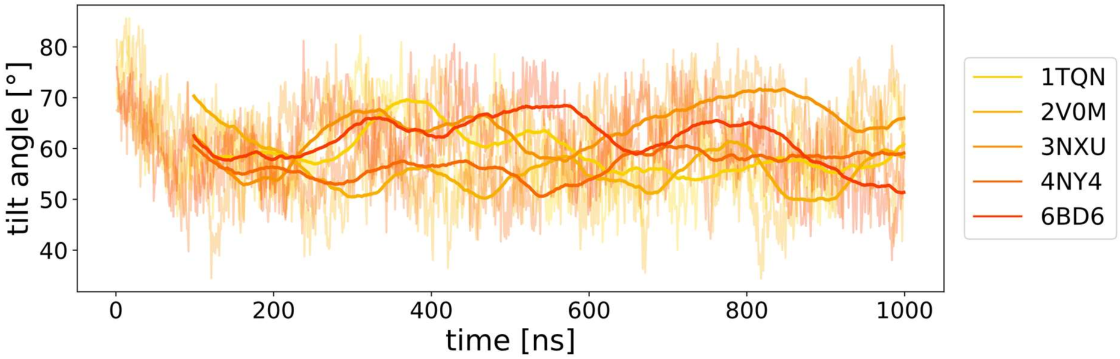

Fig. 12 tilt angles during the MD simulations. The colours are according to the underlying PDB structure. In the background with a lower transparency are the tilt angles for every ns and in the front are the rolling averages over 100 ns

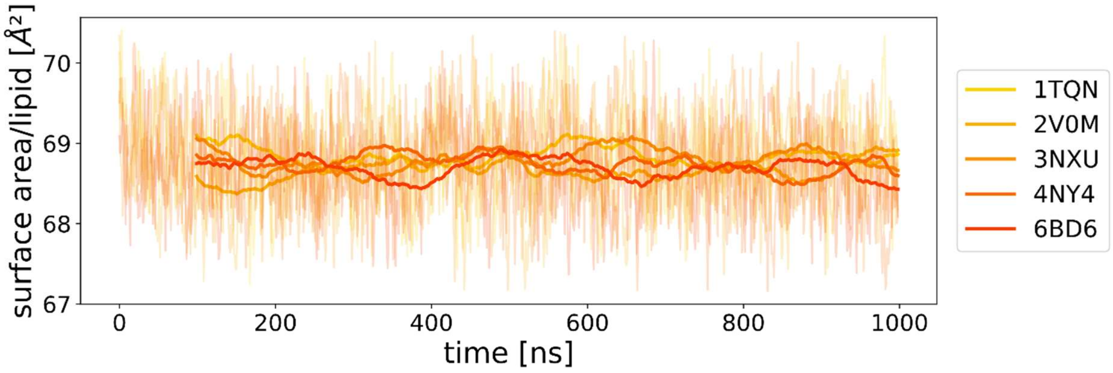

PDB code	1TQN	2V0M	3NXU	4NY4	6BD6	average
Area/lipid [$Å^2$] lower leaflet	68.9 ± 0.5	68.7 ± 0.5	68.8 ± 0.5	68.7 ± 0.5	68.7 ± 0.5	68.8 ± 0.5
Area/lipid [$Å^2$] upper leaflet	71.1 ± 0.6	71.3 ± 0.5	71.3 ± 0.6	71.2 ± 0.6	71.4 ± 0.5	71.3 ± 0.6

Fig. 13 Surface area per lipid. On top is the surface are per lipid during the simulations. The colours are according to the underlying PDB structure. In the background with a lower transparency are the surface areas per lipid for every ns and in the front are the rolling averages over 100 ns. On the bottom are the average values per simulation and the average over all 5 simulations

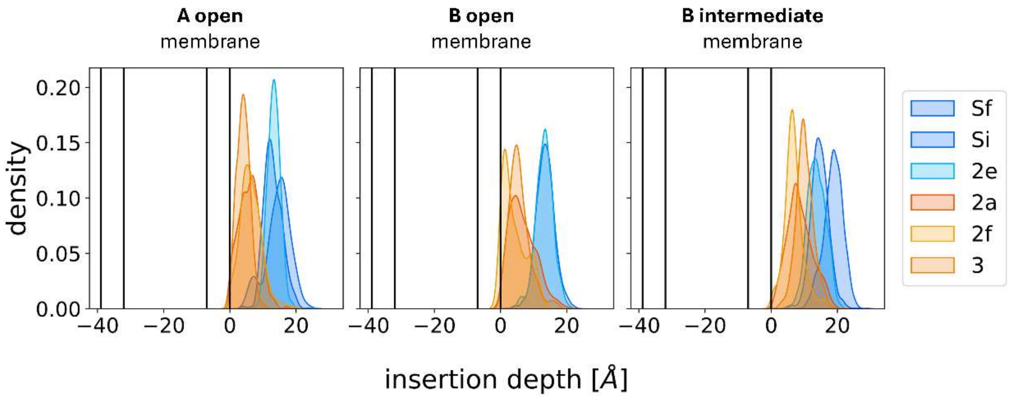

Fig. 14 Distribution of the insertion depth of the tunnel openings. The insertion depth is the distance to the polar part (nitrogen atoms of the membrane) in the upper leaflet. The colours represent the different tunnels. In blue shaded colours are the solvent facing tunnels, in orange shaded colours the membrane facing tunnels. The lines in each plot from the left to the right represent the polar part of the lower leaflet, the apolar part of the lower leaflet, the apolar part of the upper leaflet and the polar part of the upper leaflet

Table 7 Averages and standard deviations of tunnel insertion depths

Tunnel	Sf	Si	2e	2a	2f	3
Insertion depth [Å] state A open	13.1 ± 2.7	14.5 ± 3.8	13.4 ± 1.9	5.8 ± 3.1	6.4 ± 3.2	4.1 ± 1.9
Insertion depth [Å] state B open	-	13.5 ± 2.8	13.2 ± 2.6	6.7 ± 4.0	4.2 ± 3.6	5.7 ± 3.2
Insertion depth [Å] state B intermediate	18.8 ± 2.9	14.7 ± 2.7	13.7 ± 2.8	8.7 ± 3.8	6.9 ± 3.0	10.2 ± 2.6
